# Death and chemotaxis: Bacterial chemotaxis enables collective escape from phage predation

**DOI:** 10.1126/sciadv.aeg2358

**Published:** 2026-10-16

**Authors:** Victoria G. Muir, Alejandro Martínez-Calvo, Ned S. Wingreen, Sujit S. Datta

**Affiliations:** ^1^ Department of Chemical and Biological Engineering, Princeton University, Princeton, NJ 08544, USA.; ^2^ Department of Chemical and Biomolecular Engineering, University of Delaware, Newark, DE 19716, USA.; ^3^ Princeton Center for Theoretical Science, Princeton University, Princeton, NJ 08544, USA.; ^4^ Department of Chemical and Biological Engineering, Princeton University, Princeton, NJ 08544, USA.; ^5^ Department of Physics, Princeton University, Princeton, NJ 08544, USA.; ^6^ Lewis-Sigler Institute for Integrative Genomics, Princeton University, Princeton, NJ 08544, USA.; ^7^ Department of Molecular Biology, Princeton University, Princeton, NJ 08544, USA.; ^8^ Division of Chemistry and Chemical Engineering, California Institute of Technology, Pasadena, CA 91125, USA.

## Abstract

Bacteriophages (“phage”) are viruses that prey on bacteria in diverse environments, from biological tissues to soils. In many of these environments, bacterial hosts are constantly migrating, yet how bacterial migration is influenced by phage predation remains poorly understood. Using transparent granular hydrogels that mimic natural habitats, we directly visualize populations of motile *Escherichia coli* encountering lytic T4 phage. Unexpectedly, we find that even in phage-rich environments, bacteria successfully form chemotactic fronts that enable them to migrate over large distances without necessarily requiring new phage resistance mutations. At low phage concentrations, the front is indistinguishable from a phage-free control, and the population remains largely susceptible; at higher concentrations, the susceptible population initially collapses, and the front is reestablished by a less susceptible subpopulation enriched under intense predation. Higher phage concentrations delay front formation but not steady-state front speed or shape. By combining our experiments with biophysical modeling, we demonstrate that this phenomenon reflects a competition between collective chemotactic migration and phage predation, quantified by a dimensionless “escape parameter,” in which sufficiently rapid migration allows the population to outpace the spreading infection. This work thus provides mechanistic insight into how cell motility shapes phage-bacteria interactions in spatially extended environments.

## INTRODUCTION

Phage are the most abundant biological entities on Earth, shaping bacterial behavior in diverse settings ranging from the human body to terrestrial and marine ecosystems (*1*–*7*). Traditional laboratory studies examine phage-bacteria interactions in well-mixed liquid cultures, which eliminate spatial structure, or on agar plates, where the focus is typically on infection efficiency and resistance evolution (*8*–*10*). However, in nature, bacteria migrate collectively over large distances via chemotaxis (*11*, *12*), navigating chemical gradients to find nutrients, colonize hosts, cause infections, and regulate biogeochemical processes (*12*–*17*)—all while encountering phage predators (*7*). The ubiquity of this process thus presents a puzzle: How do bacteria successfully traverse these environments where phage are abundant—even outnumbering bacterial cells by up to an order of magnitude (*6*, *18*–*21*)—without being decimated?

Resolving this puzzle requires understanding the interplay between bacterial collective migration and phage predation, which remains poorly understood. Recent work has begun to address this gap in knowledge by studying how phage can “hitchhike” on migrating bacteria to copropagate through space and form characteristic spatial infection patterns with their motile hosts (*22*–*24*) and influence coevolutionary dynamics (*25*). Yet a fundamental question remains: Can bacteria traverse phage-rich environments without needing to evolve resistance, and if so, what biophysical principles govern population survival?

Here, we provide evidence that they can. Using transparent granular hydrogels that mimic natural porous habitats (*26*, *27*), we directly visualize populations of motile *Escherichia coli* encountering lytic T4 phage. By consuming chemoattractant, the bacteria establish a self-generated gradient that drives collective chemotaxis, forming migrating fronts even at the highest phage concentrations. By integrating experiments and modeling, we show that while phage continually infect and lyse a subpopulation of cells, producing “bursts” of new phage progeny, collective migration enables the population to outpace the spreading infection—directly at low phage concentrations, and, at higher concentrations, after a less susceptible subpopulation reestablishes the front—enabling long-range migration without necessarily requiring de novo evolution of resistance. This work thus reveals an ecological role of chemotaxis beyond nutrient foraging and range expansion: It enables bacteria to escape phage predators in spatially extended environments. Our findings establish quantitative principles for predicting population survival versus collapse.

## RESULTS

### Phage delay chemotactic front formation without affecting steady-state shape or speed

Our experiments explore the collective migration in glass capillaries of motile *E. coli* through granular hydrogel packings, with defined concentrations $p_{\text{init}}$ of T4 phage mixed in (Fig. 1A and Methods). Each packing is made of jammed (fig. S1), biocompatible, ∼10-μm-diameter hydrogel particles swollen in a defined rich liquid medium with glucose and L-serine as the primary nutrient and chemoattractant, respectively. The internal mesh size of each hydrogel particle is ≈40 to 100 nm (*28*), large enough to allow unimpeded transport of oxygen and nutrient (*29*–*32*) but smaller than both cells and phage (*33*). Therefore, neither cells nor phage can penetrate the individual particles; the cells are instead forced to swim through the pores formed in the interstices between particles (*26*, *34*), while the phage randomly diffuse through this pore space, remaining stable (fig. S2) and lytic (fig. S3) for at least 48 hours, much longer than the experimental duration. Moreover, because the hydrogel particles are so highly swollen with liquid, the packings are transparent—enabling us to use fluorescence confocal microscopy to directly visualize the cells, which constitutively express green fluorescent protein (GFP) in their cytoplasm, as they move in situ (*26*, *34*).

**Fig. 1.**
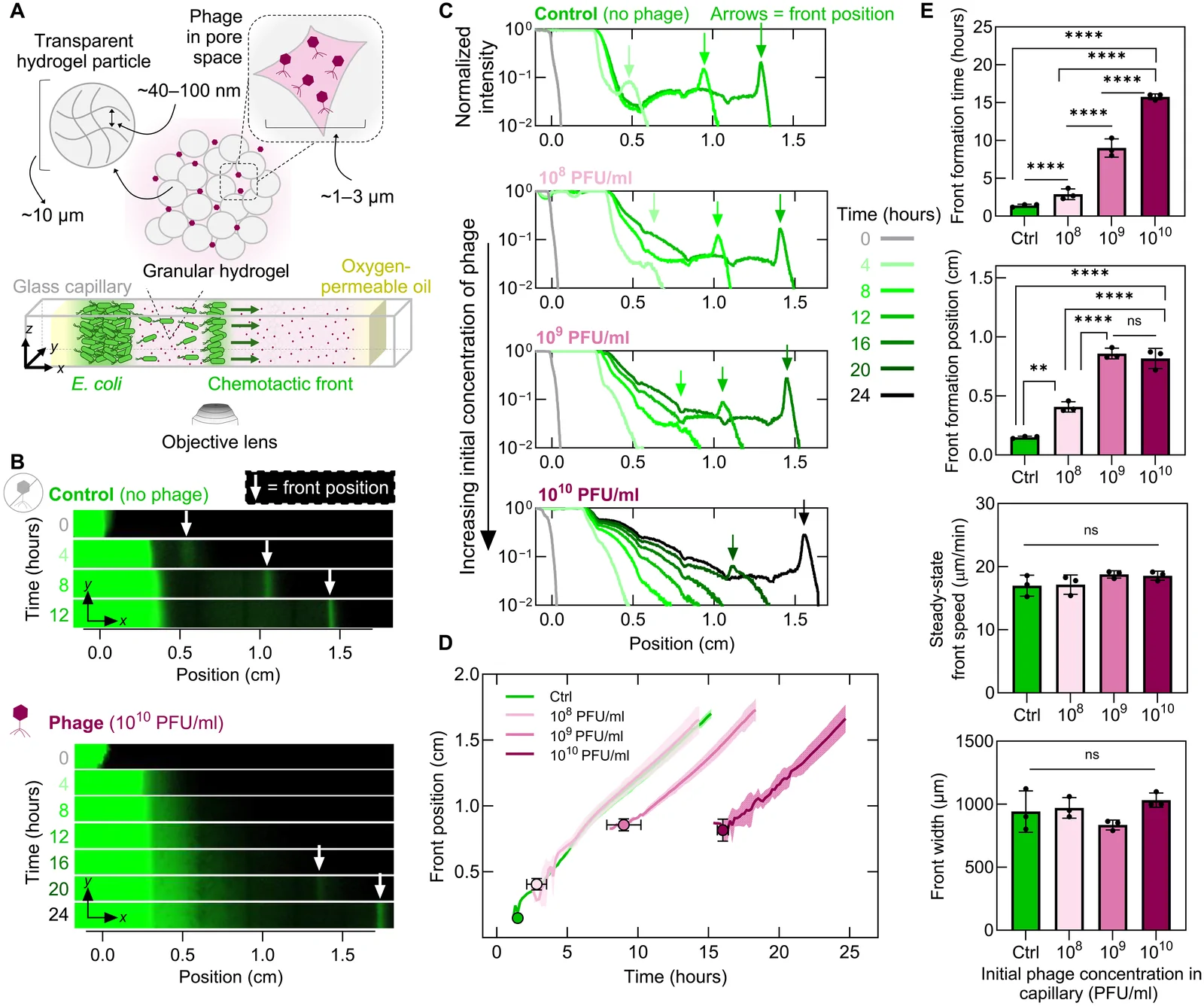
The presence of phage delays chemotactic front formation but does not alter its steady-state dynamics. (**A**) Schematic of experimental setup. Fluorescent *E. coli* migrate from a dense inoculum placed at one end of a glass capillary through the pores of a granular packing of hydrogel particles. The liquid-infused particles are transparent and permeable to nutrients and small molecules but not to bacteria or phage; therefore, phage diffuse through the pore space and infect cells. We cap each end of the capillary with oxygen-permeable paraffin oil to prevent evaporation and maintain the entire system at 30°C. (**B**) Confocal micrographs (depth *z*-integrated fluorescence signal) showing chemotactic front migration along the capillary for a phage-free control (top) and high phage concentration (bottom). (**C**) Normalized fluorescence intensity (integrated along *y* and *z*) profiles along the capillary for control, 10^8^, 10^9^, and 10^10^ PFU/ml phage concentrations. Arrows indicate front location at given times. (**D**) Chemotactic front position over time for various phage concentrations. Solid curves show average front positions; shaded regions indicate SD. Circles denote average front position at formation time; vertical and horizontal error bars show SDs. (**E**) Quantification of front formation time, formation position, steady-state speed, and width at various phage concentrations. Data represent mean ± SD (*n* ≥ 3). One-way analysis of variance (ANOVA), **P < 0.01 and ****P < 0.0001. ns, not significant.

In each experiment, we place a dense inoculum of cells [concentration $b_{\text{init}}∼10^{11}$ colony-forming units (CFU)/ml] at one end of a capillary (*x* ≈ 0) and track their migration to the other end through the hydrogel packing in real time *t* (movie S1). First, we examine the phage-free case, which serves as a negative control. The cells in the inoculum continually consume the chemoattractant, establishing a local gradient that biases their motion via chemotaxis—eventually forming a localized ∼1000-μm-wide front of migrating cells after ≈2 hours [arrows, Fig. 1, B and C (top)]. The continued consumption of chemoattractant by these cells causes the chemotactic front to continue to propagate through the capillary at a constant speed of ≈17 μm/min [green curve, Fig. 1D (top)], consistent with the findings of previous studies (*17*, *27*, *35*, *36*). The fluorescence signal intensity in the migrating front increases over time (Fig. 1B and fig. S4), reflecting the growth of new cells that compensates for leakage from the front, sustaining steady-state migration of the front with bacterial concentration *b* ∼ 10^10^ to 10^11^ CFU/ml (*37*).

Next, we repeat the same experiment but with phage uniformly dispersed through the hydrogel packings at increasing concentrations $p_{\text{init}}=10^{8}$, 10^9^, and 10^10^ plaque-forming units (PFU)/ml, corresponding to an initial effective multiplicity of infection ${\text{MOI}}_{\text{init}}\equiv p_{\text{init}}/b_{\text{init}}$ of 10^−2^ to 10^0^. These conditions approximate those found in natural environments (*6*, *18*–*21*), although unlike in well-mixed laboratory cultures, bacteria and phage are initially spatially separated here. Even under this extreme predation pressure—which rapidly and substantially suppresses population growth in well-mixed culture (fig. S3)—the bacteria form migrating chemotactic fronts in the spatially extended system, even at the highest phage concentration (Fig. 1B, bottom). At the lowest phage concentration ($p_{\text{init}}=10^{8}$ PFU/ml), the chemotactic front is indistinguishable from the phage-free control—in stark contrast to the considerable population suppression observed under the same conditions in well-mixed culture (fig. S3). The time required for each front to form and the initial position of detectable front formation both increase with phage concentration (Fig. 1E, top two). Yet, once a chemotactic front forms, it traverses the phage-rich terrain with a steady-state concentration, speed, and width that are no different from the phage-free case [Fig. 1, B to E (bottom two) and fig. S4], despite the local MOI within the front being as large as ∼10^0^, corresponding to one phage per bacterium.

### Phage exposure enriches for bacteria with reduced phage susceptibility in chemotactic fronts

Given the strong selection pressure imposed by continuous phage predation in our experiments, one possible reason why bacteria still survive and form chemotactic fronts is that the cells evolve phage resistance (*38*). To test this possibility, we isolate bacteria from chemotactic fronts after they have traversed 1 cm of the phage-laden hydrogel (Fig. 2A)—representing hours of continuous phage exposure across multiple bacterial generations. We dilute and plate these cells on soft agar that is either phage free or phage rich; each resulting CFU then originates from a single cell within the front (fig. S5).

**Fig. 2.**
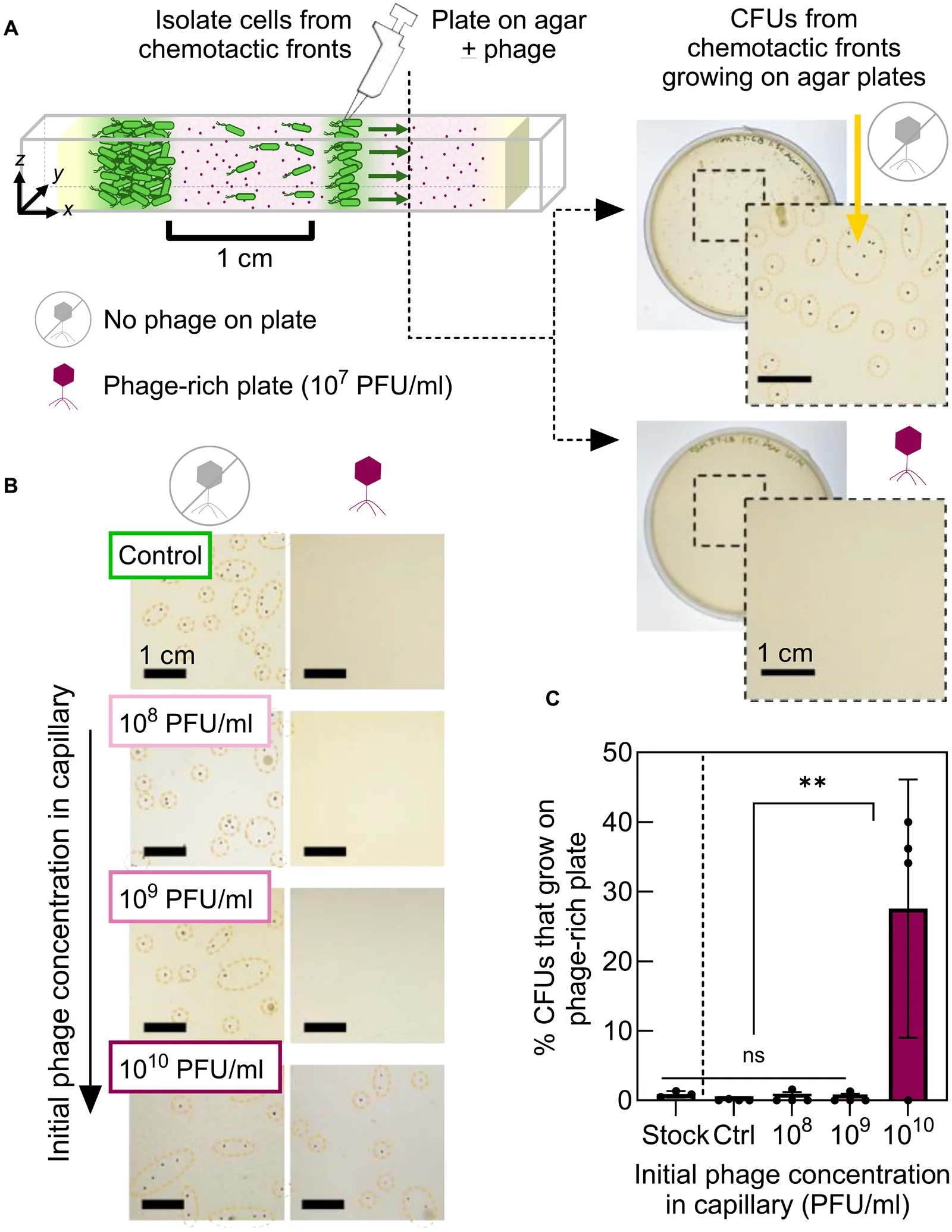
High phage exposure enriches for bacteria with reduced phage susceptibility in chemotactic fronts. (**A**) Schematic of isolating cells from chemotactic fronts after ∼1-cm migration. The cells are collected and diluted onto soft agar plates lacking phage (top) or containing phage at 10^7^ PFU/ml (bottom). Scale bars, 1 cm. (**B**) Representative images of CFUs from bacteria isolated from chemotactic fronts. Left: CFUs on phage-free plates. Right: CFUs (or absence) on phage-rich plates, depending on initial phage concentration during front formation. Scale bars, 1 cm. (**C**) Percentage of bacteria from chemotactic fronts that grow in phage-rich environment, calculated by dividing CFUs on phage-rich plates by CFUs on phage-free plates. Data represent mean ± SD (*n* ≥ 3). One-way ANOVA, **P < 0.01.

The number of CFUs on phage-free plates remains consistent across all conditions tested (Fig. 2B and fig. S5), indicating that the concentration of cells in the front is the same across experiments. None of the cells from phage-free control fronts form colonies on phage-rich plates (Fig. 2, B and C), confirming that the inoculated population lacks preexisting resistance. Moreover, cells isolated from fronts formed at the lower phage concentrations (10^8^ and 10^9^ PFU/ml) similarly cannot form colonies on phage-rich plates (Fig. 2, B and C)—indicating that, despite traversing the phage-rich terrain in a chemotactic front, most cells remain fully susceptible to phage infection. By contrast, ≈30% of cells from fronts exposed to the highest phage concentration (10^10^ PFU/ml) form colonies on phage-rich plates (Fig. 2, B and C). This finding suggests that prolonged, intense phage exposure can enrich for cells with reduced susceptibility; however, even in this extreme case, most of the cells in the migrating front remain susceptible to phage. Thus, de novo resistance evolution at the front might not be required to explain how bacterial populations traverse phage-rich environments via chemotaxis. Our data cannot, however, exclude a contribution from preexisting genetic phage-resistant variants present at low frequency in the initial inoculum (≈1% of our bacterial stock; Fig. 2C and fig. S8), which may be selectively enriched under intense phage pressure. Whether the reduced susceptibility of these front cells is genetic or purely phenotypic in origin remains to be resolved by future experiments, such as whole-genome sequencing of cells isolated from migrating fronts.

### Biophysical model reveals that collective chemotaxis enables bacteria to outrun phage bursts

If resistance does not explain bacterial survival, what does? To understand how chemotactic bacteria successfully traverse phage-rich environments, we develop a biophysical model incorporating the key features of our experimental system. All quantities are averaged along the transverse (*y*-*z*) direction, reducing the model to a one-dimensional system with space parameterized by the *x* coordinate. As summarized in Fig. 3A, we consider a population of motile, growing bacteria migrating into an initially uniform suspension of lytic phage, which are represented by the concentration field p(x,t) and have diffusivity $D_{p}$. Adsorption of phage then converts uninfected cells into infected cells (denoted by the subscript inf), which become non-motile (*39*) and lyse at a rate δ, releasing a burst of β new phage.

**Fig. 3.**
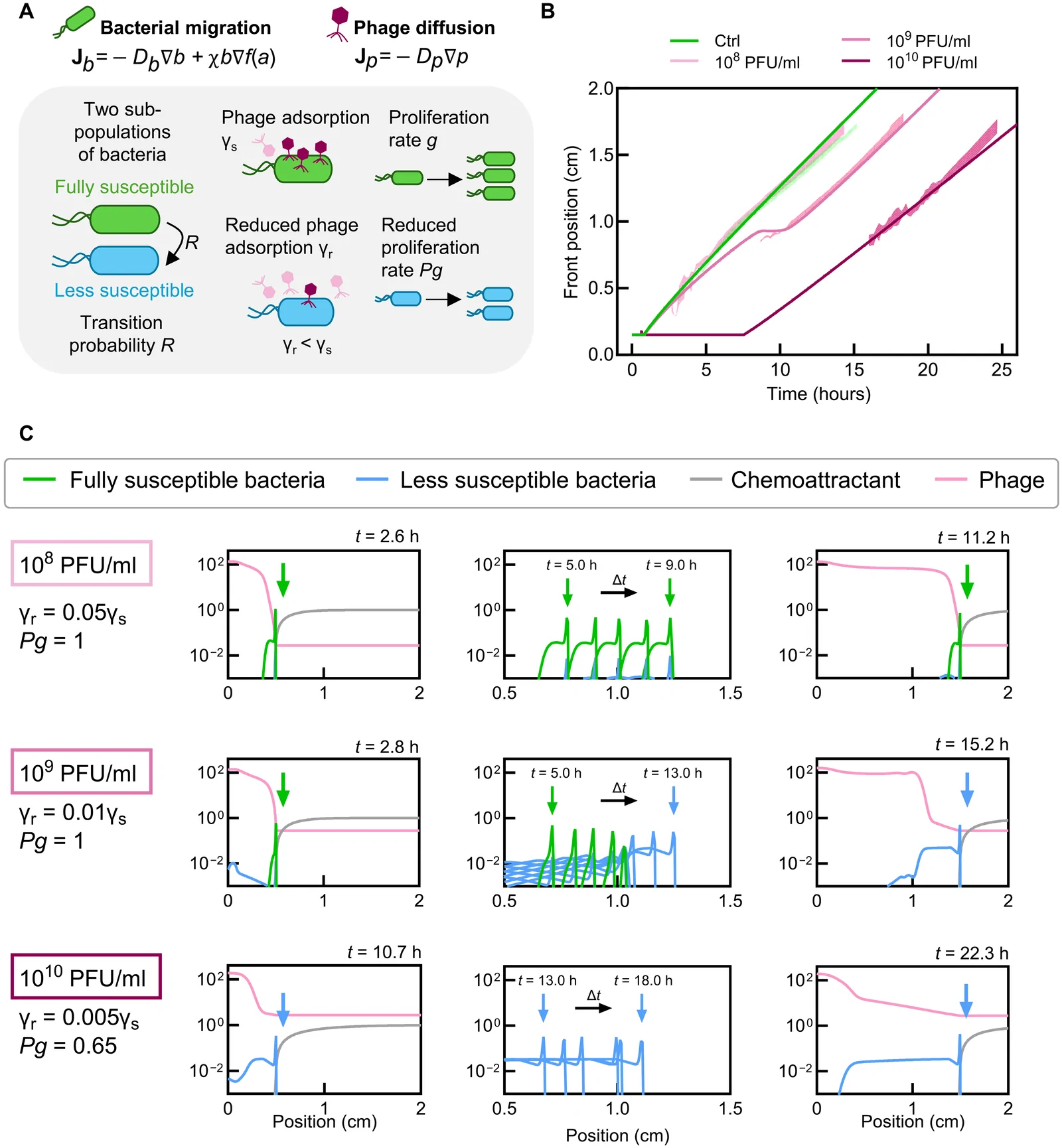
Biophysical model reproduces the essential features of bacterial collective chemotaxis in the presence of phage. (**A**) Schematic showing two swimming bacterial subpopulations: fully susceptible (growth rate *g*) and less susceptible (reduced growth rate *Pg*, *P* < 1). Fully susceptible bacteria transition to less susceptible at rate *R*. Both populations consume nutrients and perform chemotaxis identically; phage infect them at rates $\gamma_{s}$ and $\gamma_{r}<\gamma_{s}$. (**B**) Chemotactic front position versus time for phage-free environment and $p_{\text{init}}=10^{8}$, 10^9^, and 10^10^ PFU/ml, from model simulations (solid curves) and experiments (shaded regions show SD). (**C**) Normalized concentration profiles of bacteria, chemoattractant, and phage at different times for various phage concentrations. Bacteria and chemoattractant profiles are normalized by their initial concentrations at *t* = 0, and the phage profile is normalized by the characteristic concentration $\chi /\left(\gamma_{s}\ell_{c}^{2}\right)$, where $\ell_{c}$ is the chemoattractant penetration scale (Table 1 and Methods). Initially, nutrient and chemoattractant are uniform everywhere with concentrations $c_{\text{init}}$ and $a_{\text{init}}$, respectively. Bacteria are uniformly distributed in a phage-free region ($0<x<x_{\text{init}}$) adjacent to uniform phage suspension ($x_{\text{init}}<x<L$), as in the experiments. Here, $x_{\text{init}}$ marks the bacteria-phage contact interface at t=0, and *L* is the domain length. We assume that phage are stable, consistent with observations (fig. S2). At low phage concentration (10^8^ PFU/ml), bacteria rapidly establish a chemoattractant gradient and form a migrating front, leaving phage behind. At intermediate concentration (10^9^ PFU/ml), phage initially decimate the susceptible population; the less susceptible subpopulation then proliferates and reestablishes the gradient before migrating. At high concentration (10^10^ PFU/ml), this delay is prolonged, but the front eventually forms and migrates at the same steady-state speed. Arrows indicate chemotactic front position.

Bacterial populations naturally exhibit heterogeneity in susceptibility to phage, arising from purely phenotypic variation in receptor expression and phage adsorption rates (*40*–*42*). To capture this variability in a simplified manner, we classify the bacteria into two subpopulations $b_{s}\left(x,t\right)$ and $b_{r}\left(x,t\right)$: fully susceptible cells with infection rate $\gamma_{s}$ (99% of the initial population) and less susceptible cells with a reduced infection rate $\gamma_{r}<\gamma_{s}$ (1%) due to reduced phage adsorption, respectively. Motivated by our experimental findings and by prior work on phenotypic heterogeneity in phage susceptibility (*40*, *41*), we model the less susceptible subpopulation as preexisting in the initial inoculum at 1% frequency (consistent with fig. S8) rather than arising from de novo mutations during migration, although our experiments cannot rule out the latter. In addition, to capture phenotypic plasticity in phage susceptibility (*43*–*45*), we include a small transition rate *R* for susceptible cells to become less susceptible. We also incorporate a growth penalty P∈(0,1) for less susceptible cells to describe the fitness cost associated with reduced phage susceptibility (*46*, *47*). The value P=0.65 is set by the measured biomass accumulation of cells isolated from high-phage fronts (fig. S7), which is reduced by ≈60% relative to controls. Furthermore, $\gamma_{r}$ is fitted independently for each phage concentration; direct measurement via controlled infection assays on the isolated less susceptible subpopulation is a useful direction for future work.

Both bacterial subpopulations consume nutrient c(x,t), which has diffusivity $D_{c}$ and enables the cells to grow at a maximal rate *g* modulated by the Michaelis-Menten function $f_{g}\left(c\right)=c/\left(c+c_{M}\right)$ with characteristic concentration $c_{M}$. They also consume chemoattractant a(x,t), which has diffusivity $D_{a}$, creating a self-generated gradient that drives chemotactic migration with flux $J_{i}=-D_{b}\nabla b_{i}+b_{i}v_{\text{chemo}}$. The first term describes unbiased random motion of cells with effective diffusivity $D_{b}$, and the second describes their directed chemotaxis with velocity $v_{\text{chemo}}=\chi \nabla f_{a}\left(a\right)$. Here, $f_{a}\left(a\right)=\text{ln}\left[\left(1+a/a_{-}\right)/\left(1+a/a_{+}\right)\right]$ describes the ability of the bacteria to logarithmically sense the chemoattractant (*17*, *35*, *36*, *48*, *49*) with upper and lower characteristic concentrations $a_{+}$ and $a_{-}$, respectively, and the chemotactic coefficient χ describes their ability to move up the sensed gradient. We assume that both nutrient and chemoattractant are consumed at a maximal rate *k*, also following the same Michaelis-Menten kinetics as growth. Thus, in our model, growth and directed migration are only partially coupled; biomass accumulation is governed by consumption of the nutrient *c*, whereas front propagation is driven by the self-generated gradient in the chemoattractant *a*.

Together, these processes are summarized by the following dynamical equations:

Susceptible bacteria

$$\partial_{t}b_{s}=\underset{\text{Diffusion }\&\;\text{chemotaxis}}{\underbrace{-\nabla \cdot J_{s}}}+\underset{\text{Growth}}{\underbrace{\left(1-R\right){\mathrm{gb}}_{s}f_{g}\left(c\right)}}-\underset{\text{Infection}}{\underbrace{\gamma_{s}b_{s}p}}$$ (1)

Infected susceptible bacteria

$$\partial_{t}b_{s,\text{inf}}=\underset{\text{Infection}}{\underbrace{\gamma_{s}b_{s}p}}-\underset{\text{Death}}{\underbrace{\delta b_{s,\text{inf}}}}$$ (2)

Less susceptible bacteria

$$\partial_{t}b_{r}=\underset{\text{Diffusion }\&\;\text{chemotaxis}}{\underbrace{-\nabla \cdot J_{r}}}+\underset{\text{Growth}}{\underbrace{\left[{\mathrm{Rb}}_{s}+{\mathrm{Pb}}_{r}\right]{\mathrm{gf}}_{g}\left(c\right)}}-\underset{\text{Infection}}{\underbrace{\gamma_{r}b_{r}p}}$$ (3)

Infected less susceptible bacteria

$$\partial_{t}b_{r,\text{inf}}=\underset{\text{Infection}}{\underbrace{\gamma_{r}b_{r}p}}-\underset{\text{Death}}{\underbrace{\delta b_{r,\text{inf}}}}$$ (4)

Phage

$$\begin{array}{c} \partial_{t}p=\underset{\text{Diffusion}}{\underbrace{D_{p}{\text{∇}}^{2}p}}-\\ \underset{\text{Adsorption}}{\underbrace{\gamma_{s}\left(b_{s}+b_{s,\text{inf}}\right)p-\gamma_{r}\left(b_{r}+b_{r,\text{inf}}\right)p}}+\underset{\text{Lysis and bursting}}{\underbrace{\beta \delta \left(b_{s,\text{inf}}+b_{r,\text{inf}}\right)}} \end{array}$$ (5)

Nutrient

$$\partial_{t}c=\underset{\text{Diffusion}}{\underbrace{D_{c}{\text{∇}}^{2}c}}-\underset{\text{Uptake}}{\underbrace{k\left(b_{s}+b_{r}\right)f_{g}\left(c\right)}}$$ (6)

Chemoattractant

$$\partial_{t}a=\underset{\text{Diffusion}}{\underbrace{D_{a}{\text{∇}}^{2}a}}-\underset{\text{Uptake}}{\underbrace{k\left(b_{s}+b_{r}\right)f_{g}\left(a\right)}}$$ (7)

where we have neglected the contribution of chemoattractant uptake to growth since c≫a.

### Model recapitulates experimental observations of front formation and steady-state dynamics

We solve Eqs. 1 to 7 numerically, with no-flux boundary conditions for all fields and initial conditions matching the experiments (Table 1 and figs. S6 to S9). As shown in Fig. 3 (B and C) and movies S2 to S4, the simulations, despite their simplicity, recapitulate all the key features of the experimental observations: (i) delays in chemotactic front formation that increase with phage concentration, (ii) steady-state front speeds and widths that remain unchanged despite phage predation, and (iii) sustained bacterial concentration within migrating fronts across all phage concentrations.

**Table 1.** Estimates and measurements of the parameter values of the model.

Physical parameters	Definition	Range of values	Source
ϕ	Void hydrogel volume fraction	0.17	Ref. (*36*)
$b_{s,\text{init}}$	Initial susceptible bacterial density	7 × 10^10^ cells ml^−1^	Measured
$b_{r,\text{init}}$	Initial less susceptible bacterial density	1% $b_{s,\text{init}}$	Measured
$p_{\text{init}}$	Initial phage density	10^8^ − 10^10^ phage ml^−1^	Measured
$c_{\text{init}}$	Initial carbon source concentration	20 mM	Refs. (*29*, *36*) and measured
$a_{\text{init}}$	Initial chemoattractant concentration	10 mM	Refs. (*29*, *36*) and measured
$x_{\text{init}}$	Initial thickness of bacterial inoculum	1 mm	Measured
*g*	Maximal bacterial growth rate	0.9 hour^−1^	Measured
$k_{c}=k_{a}=k$	Bacterial maximal uptake rate (nutrient and chemoattractant)	7.8 × 10^−11^ μmol (min cell)^−1^	Ref. (*29*)
$c_{M}$	Michaelis-Menten half-velocity constant (nutrient and chemoattractant)	3 μM	Refs. (*29*, *35*, *36*)
$\left(a_{-},a_{+}\right)$	Characteristic sensing concentrations	(8,200) μM	Refs. (*35*, *36*, *49*)
$D_{b}$	Bacteria diffusivity	1.2 μm^2^ s^−1^	Measured
$D_{c}=D_{a}=D$	Nutrient and chemoattractant diffusivity	800 μm^2^ s^−1^	Refs. (*29*, *35*, *36*)
$D_{p}$	Phage diffusivity	11 μm^2^ s^−1^	Measured
χ	Chemotactic strength	16 μm^2^ s^−1^	Ref. (*36*) and fitted
$\gamma_{s}$	Adsorption rate (susceptible)	3 × 10^−12^ ml phage^−1^ min^−1^	Ref. (*22*) and fitted
$\gamma_{r}$	Adsorption rate (less susceptible)	5 × 10^−3^ − 10^−2^ γ_s_	Fitted
β	Burst size	90	Refs. (*51*–*55*) and fitted
δ	Bacterial death rate	1 hour^−1^	Refs. (*53*–*55*)
*R*	Susceptible → less susceptible rate	10^−3^	Ref. (*22*)
*P*	Growth penalty	0.65 − 1	Ref. (*22*) and fig. S7
Characteristic scales
$\ell_{c}=\sqrt{{\mathrm{Da}}_{\text{init}}/\left({\mathrm{kb}}_{s,\text{init}}\right)}$	Characteristic length scale	296.5 μm	
$t_{c}=\ell_{c}^{2}/\chi$	Characteristic timescale	1.53 hour	
$b_{c}=b_{s,\text{init}}$	Characteristic bacterial density (all bacterial fields)	7 × 10^10^ cells ml^−1^	
$p_{c}={\left(\gamma_{s}t_{c}\right)}^{-1}=\chi /\left(\gamma_{s}\ell_{c}^{2}\right)$	Characteristic phage density scale	3.64 × 10^9^ phage ml^−1^	
$c_{c}=c_{\text{init}}$	Characteristic carbon source concentration scale	20 mM	
$a_{c}=a_{\text{init}}$	Characteristic chemoattractant concentration scale	10 mM	
Dimensionless parameters
$\tilde{\chi }\equiv \left(\chi /\ell_{c}^{2}\right)/\left(b_{s,\text{init}}\gamma_{s}\right)$	Escape parameter	0.05	
β	Burst size	90	
$\tilde{\delta }\equiv \delta t_{c}$	Bacterial death rate/bacterial chemotaxis rate	1.53	
$\tilde{g}\equiv {\mathrm{gt}}_{c}=g/\left(\chi /\ell_{c}^{2}\right)$	Bacterial growth rate/bacterial chemotaxis rate	1.38	
$\text{MOI}\equiv p_{\text{init}}/\left(b_{s,\text{init}}\right)$	Multiplicity of infection	10^−3^ − 10^−1^	
$\gamma_{r}/\gamma_{s}$	Less susceptible–to–susceptible adsorption ratio	5 × 10^−3^ − 10^−2^	
$b_{r,\text{init}}/b_{s,\text{init}}$	Less susceptible–to–susceptible initial density	10^−2^	
*R*	Susceptible → less susceptible rate	10^−3^	
*P*	Growth penalty	0.65 − 1	
$a_{\text{init}}/c_{\text{init}}$	Ratio of initial concentrations	0.5	
${\tilde{D}}_{b}\equiv D_{b}/\chi$	Bacterial diffusivity/bacterial chemotaxis	0.075	
${\tilde{D}}_{p}\equiv D_{p}/\chi$	Phage diffusivity/bacterial chemotaxis	0.69	
$\Gamma_{c}\equiv \chi /D$	Bacterial chemotaxis/nutrient diffusivity	0.02	
$\Gamma_{a}\equiv \Gamma_{c}\equiv \Gamma$	Bacterial chemotaxis/chemoattractant diffusivity	0.02	
${\tilde{c}}_{M,c}\equiv c_{M}/c_{\text{init}}$	Dissociation constant/initial concentration	1.5 × 10^−4^	
${\tilde{c}}_{M,a}\equiv c_{M}/a_{\text{init}}$	Dissociation constant/initial concentration	3 × 10^−4^	
${\tilde{x}}_{\text{init}}\equiv x_{\text{init}}/\ell_{c}$	Dimensionless thickness of bacterial inoculum	3.37	

At the lowest phage concentration ($p_{\text{init}}=10^{8}$ PFU/ml, ${\text{MOI}}_{\text{init}}∼$), the bacterial population has sufficient time to consume nutrients, proliferate, and establish a strong chemoattractant gradient before phages can diffuse into the bacterial population and begin killing cells (Fig. 3C and movie S2). The resulting chemotactic migration is fast enough to prevent the spread of infection from newly produced phage, which are left behind the advancing front (pink curve in Fig. 3C, rightmost). Consequently, the steady-state front speed, width, and bacterial concentration remain unchanged despite phage predation (Fig. 3, B and C), in good agreement with the experimental observations.

As phage concentration increases to $p_{\text{init}}=10^{9}$ and 10^10^ PFU/ml (${\text{MOI}}_{\text{init}}∼10^{-1}$ and 10^0^, respectively), phage predation initially outpaces bacterial chemotaxis, leading to collapse of the fully susceptible subpopulation (Fig. 3C and movies S3 and S4). The less susceptible bacteria must then proliferate to a high concentration and reestablish a chemoattractant gradient before the front can migrate, causing both the time and position of front formation to increase with phage concentration (Fig. 3B). However, once fronts form, they propagate at the same steady-state speed as the phage-free control across all conditions tested, in good agreement with our experimental findings. The magnitude of this delay depends on the absolute number of less susceptible cells in the initial inoculum: Additional simulations show that raising their initial fraction or enlarging the inoculum reduces the delay, while a smaller inoculum lengthens it (figs. S11 and S12). The effect strengthens with phage concentration, where front formation relies more critically on the less susceptible subpopulation to rebuild the chemoattractant gradient after the susceptible subpopulation collapses—consistent with the delay being set by the time for this rare subpopulation to proliferate to a front-forming density, which depends only weakly on its initial number. In all of these additional simulations, the steady-state front speed remains unchanged.

These results demonstrate that our minimal biophysical model quantitatively recapitulates the key experimental observations and unveils the underlying mechanism: Collective migration enables the population to outpace the spreading infection. At low phage concentrations, the largely susceptible population outpaces the infection on its own; at high concentrations, it collapses, and a less susceptible subpopulation proliferates and reestablishes the migrating front. Collective migration through a spatially extended environment thus enables population persistence, in stark contrast to well-mixed populations where the same phage concentrations rapidly and substantially suppress the susceptible population. A notable difference between the model and experiments emerges at the highest phage concentration, 10^10^ PFU/ml: In the model, the fully susceptible subpopulation collapses almost completely before the less susceptible subpopulation reestablishes the front, so that the front becomes almost entirely less susceptible, while in the experiments, the fronts remain mixed, with ≈70% of cells still susceptible and only ∼30% showing reduced susceptibility (Fig. 2C). This discrepancy likely reflects simplifications inherent to our two-subpopulation description—in particular, the discrete (rather than continuous) representation of susceptibility and the fixed susceptible-to-less-susceptible transition rate *R*, which together may overestimate the extent of susceptible-population collapse under intense phage predation.

### Dimensionless escape parameter predicts front formation dynamics

Our simulations show that whether bacteria can outrun phage depends on the competition between chemotactic migration and phage infection. To quantify this competition, we compare their characteristic rates. Bacterial chemotaxis occurs at rate $∼\chi /\ell_{c}^{2}$, where χ is the chemotactic coefficient and $\ell_{c}=\sqrt{\frac{D_{a}a_{\text{init}}}{{\mathrm{kb}}_{s,\text{init}}}}$ is the characteristic distance over which chemoattractant is depleted by the migrating front, which sets the front width. Phage infection depletes the bacterial population at rate $∼\gamma_{s}b_{s,\text{init}}$, determined jointly by the efficacy of phage adsorption and the density of bacteria. The ratio of these two rates defines a dimensionless escape parameter, $\tilde{\chi }\equiv \left(\chi /\ell_{c}^{2}\right)/\left(\gamma_{s}b_{s,\text{init}}\right)=\mathrm{k\chi}/\left(\gamma_{s}D_{a}a_{\text{init}}\right)$. When $\tilde{\chi }\gg 1$, we expect that bacteria can escape phage infection using chemotaxis; conversely, when $\tilde{\chi }\ll 1$, infection outpaces chemotaxis, and front formation is increasingly delayed. Whether bacteria ultimately survive also depends on the initial effective multiplicity of infection ${\text{MOI}}_{\text{init}}\equiv p_{\text{init}}/b_{\text{init}}$; when ${\text{MOI}}_{\text{init}}$ is large, we expect that phage rapidly decimate the susceptible population before a chemotactic gradient can be established, while when ${\text{MOI}}_{\text{init}}$ is small, bacteria have sufficient time to proliferate and form a migrating front before widespread infection occurs. While other parameters, such as the phage burst size β (fig. S10), can also influence front formation dynamics, we focus on $\tilde{\chi }$ and ${\text{MOI}}_{\text{init}}$ because these two dimensionless parameters are directly controllable in our experiments and capture the essential processes underlying chemotactic front formation. To determine how $\tilde{\chi }$ and the initial multiplicity of infection ${\text{MOI}}_{\text{init}}$ jointly dictate population behavior, we perform 58 different simulations across a wide range of parameter values (Table 1).

The resulting state diagram (Fig. 4) reveals three distinct regimes. At high $\tilde{\chi }$ and low ${\text{MOI}}_{\text{init}}$ (faster chemotaxis, fewer phage), fronts form immediately without delay (Fig. 4, light gray), indistinguishable from the phage-free control—bacterial chemotaxis is so rapid that phage predation has negligible impact. At intermediate conditions, fronts exhibit delays in formation (medium gray) that increase with ${\text{MOI}}_{\text{init}}$, as the less susceptible subpopulation must proliferate and reestablish the chemotactic gradient after the susceptible population collapses; however, once formed, these fronts propagate at the same steady-state speed as the control case. Our three experimental conditions ($p_{\text{init}}=10^{8}$, 10^9^, and 10^10^ PFU/ml at χ = 16 μm^2^/s) map directly onto these regimes, as shown by the pink circles in Fig. 4. Ultimately, at sufficiently low $\tilde{\chi }$ and high ${\text{MOI}}_{\text{init}}$ (slower chemotaxis, abundant phage), we expect that phage predation outpaces chemotaxis, preventing front formation entirely—the front formation time diverges and the migrating population collapses.

**Fig. 4.**
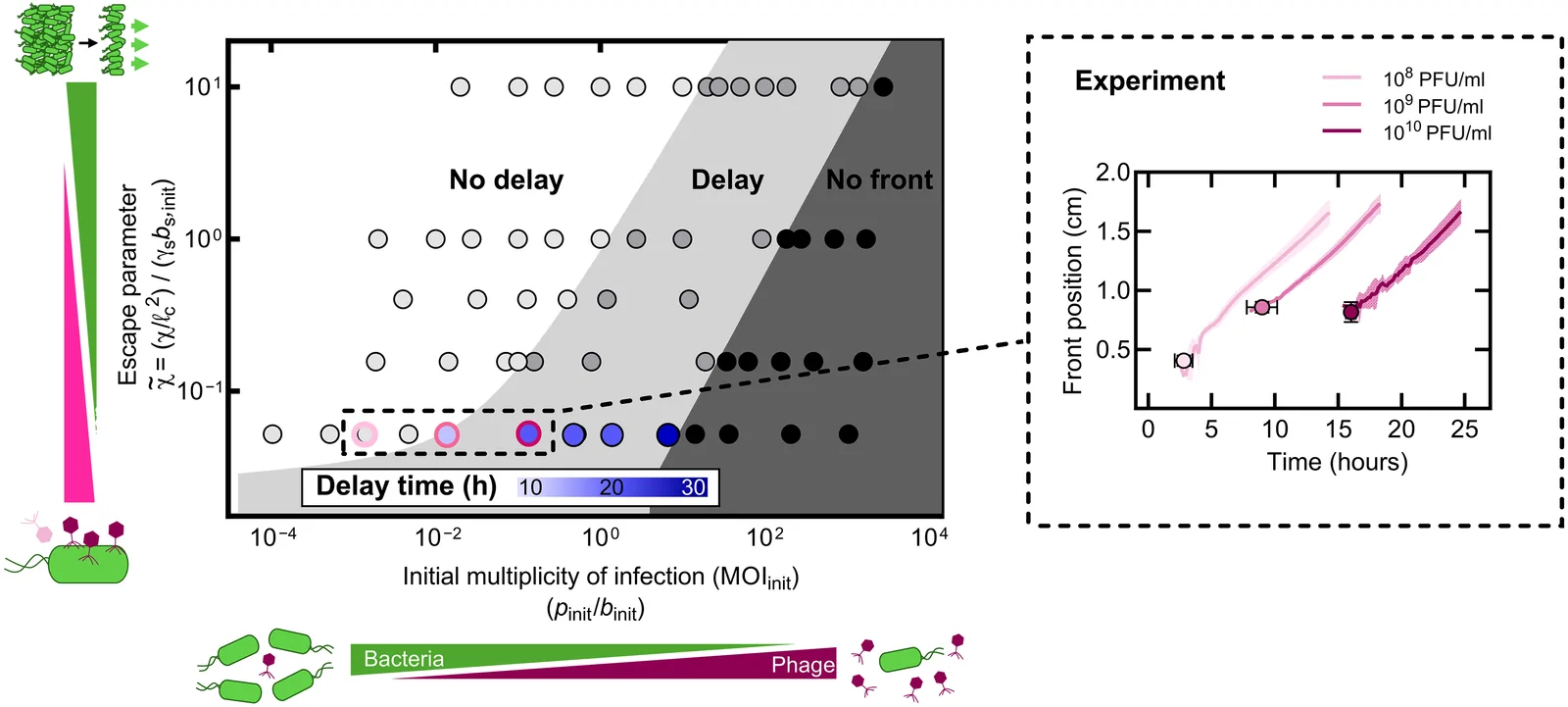
State diagram shows how the escape parameter $\tilde{\chi }$ and initial multiplicity of infection ${\text{MOI}}_{\text{init}}$ influence chemotactic front formation timing. State diagram from biophysical model simulations showing how chemotaxis, phage predation, and initial phage-to-bacteria ratio affect front formation. Vertical axis: dimensionless escape parameter $\tilde{\chi }\equiv \left(\chi /\ell_{c}^{2}\right)/\left(\gamma_{s}b_{s,\text{init}}\right)$, which compares the rates of chemotaxis and phage infection; $\ell_{c}$ is the distance over which chemoattractant penetrates into the migrating front. Horizontal axis: initial multiplicity of infection ${\text{MOI}}_{\text{init}}=p_{\text{init}}/b_{s,\text{init}}$. As ${\text{MOI}}_{\text{init}}$ increases, phage predation increasingly delays front formation, eventually preventing formation entirely. As $\tilde{\chi }$ increases, bacteria increasingly outrun phage via enhanced chemotactic efficiency, reducing delays. While other parameters of the model, such as the phage burst size or bacterial lysis time, can affect the position of the boundaries delimiting these regions, we expect the same qualitative behavior. The boundary delimiting the no delay-delay regions is well described by the following empirical function: $0.05\;{\text{MOI}}_{\text{init}}^{1/20}+0.8\;{\text{MOI}}_{\text{init}}^{9/10}$, whereas the boundary delimiting the delay-no front regions is approximately described by the empirical scaling $0.006\;{\text{MOI}}_{\text{init}}$. For a fixed value of $\tilde{\chi }$ = 0.05, the delay time predicted by model simulations is shown as the blue-shaded regions. Light pink circle: experimental condition at 10^8^ PFU/ml showing minimal delay. Medium and dark pink circles: conditions at 10^9^ and 10^10^ PFU/ml showing increasing delays. Inset: front position over time for three experimental datasets highlighted in phase diagram and shown in Figs. 1D and 3B. Data represent mean ± SD (*n* ≥ 3).

The hydrogel platform provides a straightforward way to further test this framework: Reducing the packing density of hydrogel particles increases the sizes of the pores between them, increasing chemotactic efficiency purely physically by reducing cellular confinement (*27*, *36*, *50*). Therefore, as a final test of the model, we repeat the experiment for $p_{\text{init}}=10^{9}$ PFU/ml but in looser hydrogel packings, for which χ increases from 16 to 40 μm^2^/s (movie S5). The theory predicts that, in this case, a chemotactic front should form without delay (Fig. 5A), with no influence of phage predation on front formation time or steady-state speed. Our experiments directly confirm this prediction, as shown in Fig. 5 (B to D), providing additional validation of our biophysical framework. Together, these results establish our state diagram as a quantitative framework for predicting bacterial survival: For a given ${\text{MOI}}_{\text{init}}$, increasing $\tilde{\chi }$, either by improving chemotactic efficiency or hindering phage infection, enables bacteria to collectively escape phage predation.

**Fig. 5.**
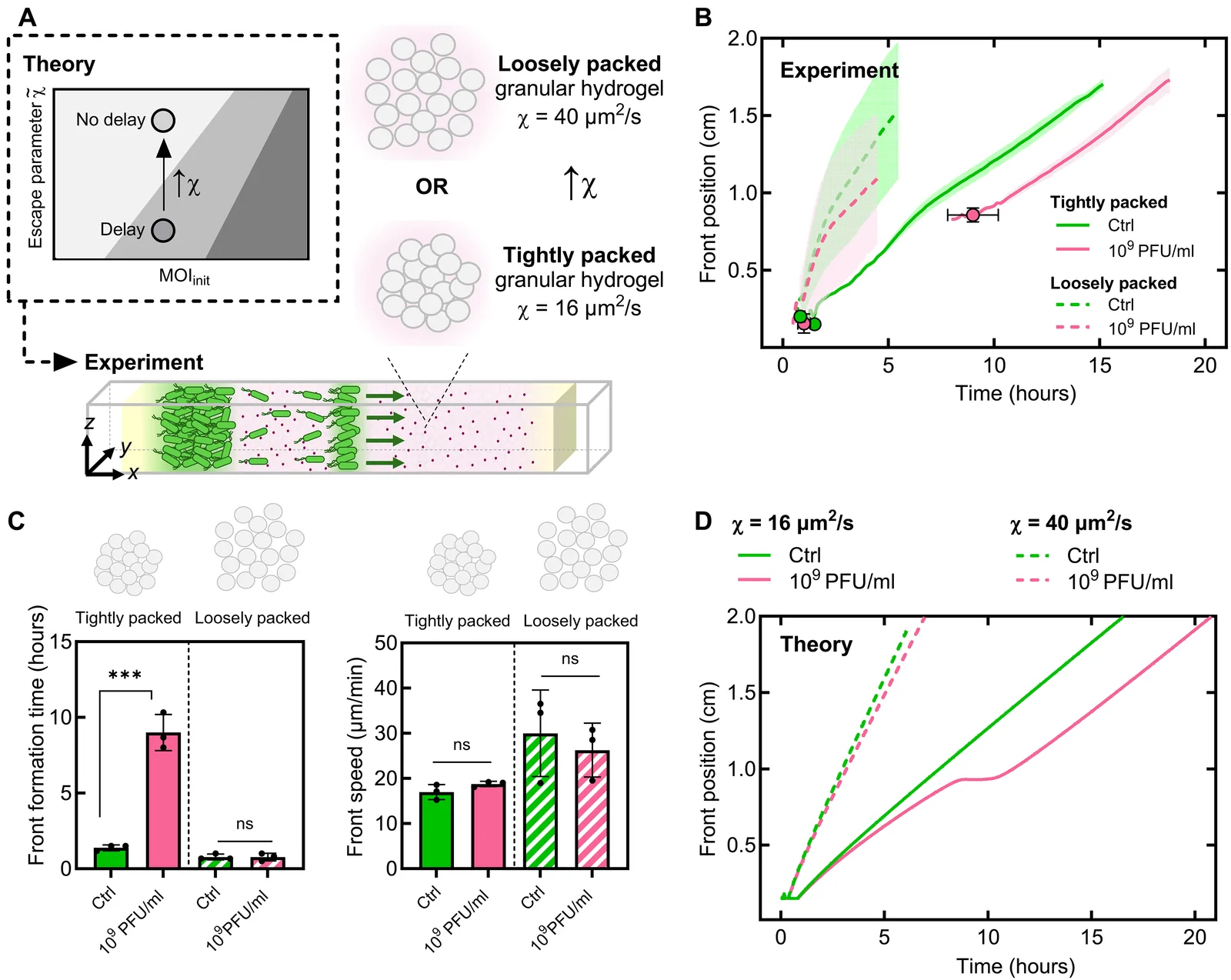
Increasing chemotactic efficiency enables escape from phage predation. (**A**) Schematic showing how decreasing the hydrogel particle packing density improves bacterial chemotactic efficiency; our theory predicts that, in this case, chemotactic front formation will no longer be delayed. (**B**) Bacterial front position over time in tightly versus loosely packed hydrogels, either for the no-phage control (green) or when loaded with 10^9^ PFU/ml phage (pink). (**C**) Front formation time and steady-state speed without phage (control) and with 10^9^ PFU/ml phage for tightly and loosely packed hydrogels. (**D**) Theoretical predictions for the same χ values as experiments. Model predicts no delay at χ = 40 μm^2^/s versus 9-hour delay at χ = 16 μm^2^/s. Data represent mean ± SD (*n* ≥ 3). One-way ANOVA, ***P < 0.001.

## DISCUSSION

Our work helps to resolve the puzzle of how bacteria successfully traverse phage-rich terrain, from the human gut to ocean sediments and agricultural soils, without being decimated. We show that collective chemotactic migration enables bacteria to escape phage predation in spatially extended environments by outpacing the spreading infection (Fig. 6), without requiring de novo evolution of genetic resistance—a finding that challenges the prevailing focus on development of resistance as the primary survival mechanism, a paradigm established in well-mixed liquid cultures where resistance mutations consistently emerge and spread. The resulting chemotactic-escape picture depends on phage concentration: At low predation pressure, the bulk of the susceptible population migrates fast enough to outpace the spreading infection, while at higher phage concentrations, intense predation selectively enriches a small preexisting low-susceptibility subpopulation that reestablishes the chemotactic front, enabling the population to persist and traverse the phage-rich environment. The genetic versus phenotypic nature of this subpopulation and the possible emergence of de novo phage resistance during migration are important open questions to address in future work. Moreover, our biophysical model provides a framework that unifies the different effects of bacterial motility, chemoattractant consumption and sensing, phage infection, and the abundance of both phage and bacteria, yielding quantitative principles to predict and control these dynamics more broadly.

**Fig. 6.**
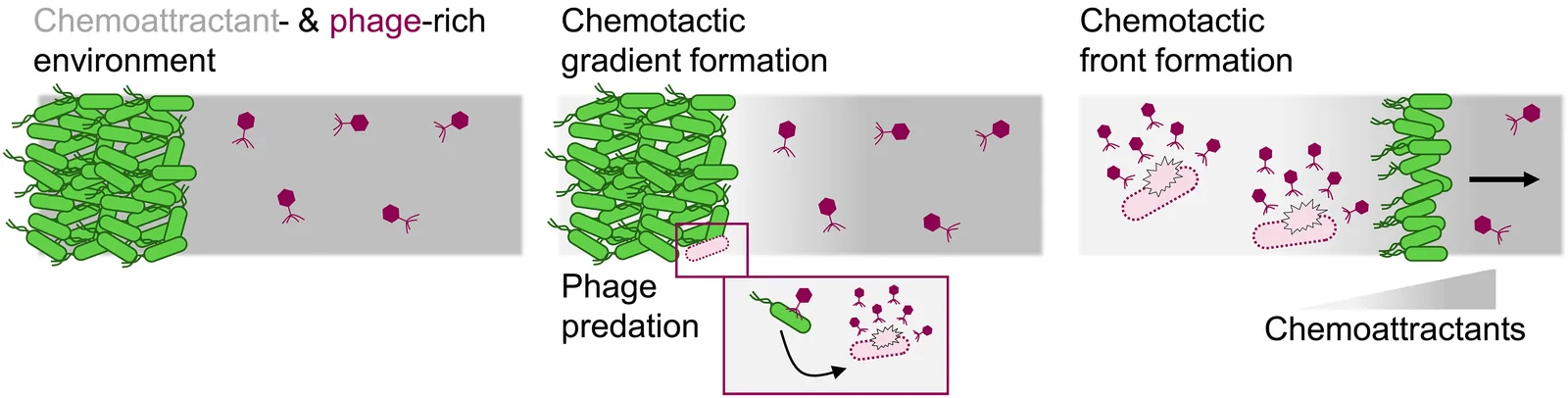
Schematic overview of findings. Bacteria inoculated at the left edge of a phage-rich environment (left) consume chemoattractant and establish a self-generated gradient (middle). Despite continuous phage infection and lysis of cells within the population, collective chemotactic migration up this gradient is fast enough to outrun the trailing burst of phage progeny, enabling the formation and propagation of a migrating chemotactic front (right). This mechanism allows bacterial populations to successfully traverse phage-rich terrain over large distances without requiring evolution of genetic resistance.

Several simplifications in the model represent opportunities for future investigation:

1) We assumed that cells transition from susceptible to less susceptible at a fixed rate, but in reality, phenotypic transitions may also depend on environmental conditions including local phage concentration, nutrient availability, and population density. Incorporating such context-dependent transitions could reveal how bacteria dynamically modulate their susceptibility in response to infection pressure.

2) We classified bacteria into just two discrete subpopulations, but natural populations exhibit continuous distributions of susceptibility arising from cell-to-cell heterogeneity in receptor expression and physiological state. Extending the model to include a spectrum of susceptibility states, or stochastic transitions between states, would provide a more realistic representation of population-level dynamics.

3) Less susceptible cells may carry modified surface receptors, e.g., truncated LPS or altered outer-membrane proteins, that allow them avoid being infected by adsorbed phage—instead, potentially acting as local sinks that deplete free phage and shield coexisting susceptible cells. However, in our experiments with $\gamma_{r}\approx 5\times 10^{-3}\gamma_{s}$ and $b_{r}/b_{s}\approx 0.3/0.7$, the fraction of phage removed by the less susceptible subpopulation is $\gamma_{r}b_{r}/\left(\gamma_{s}b_{s}+\gamma_{r}b_{r}\right)∼0.2\%$, far too little to shield the susceptible cells. Adsorption assays could shed further light on this possibility by providing measurements of γ_r_ in such mixed populations, alongside sequencing of cells isolated from the front to establish whether reduced susceptibility is genetic or phenotypic in origin.

4) While we described front formation dynamics using the experimentally tunable dimensionless parameters $\tilde{\chi }$ and ${\text{MOI}}_{\text{init}}$, other parameters also influence population survival. For example, phage burst size β determines how rapidly phage populations amplify following infection (fig. S10), while bacterial growth rate *g* affects how quickly the population can recover from infection events. Thus, alternative dimensionless groups may provide additional physical insight.

5) While $\tilde{\chi }$ provides a useful organizing framework for understanding the competition between chemotaxis and phage infection, whether this framework holds quantitatively when the physical parameters that $\tilde{\chi }$ depends on are varied independently remains to be experimentally validated. For instance, $\tilde{\chi }$ is independent of the initial bacterial density, while other dimensionless parameters that govern the phase boundaries in Fig. 4 do depend on it. Exploring these parametric dependencies will be a useful direction for future research.

Experimentally, our findings motivate new approaches to directly assessing phenotypic changes during migration. RNA sequencing or single-cell transcriptomics at defined spatial positions along chemotactic fronts could identify molecular signatures associated with transient or more permanent susceptibility changes. Time-lapse imaging of individual cells expressing fluorescent phage-receptor proteins could reveal how receptor expression varies spatially within migrating fronts and whether this variation correlates with infection probability. Last, while our experiments used *E. coli* and T4 phage as a model system, our transparent hydrogel platform enables study of diverse phage-bacteria pairs across different ecological contexts, from pathogens in clinical settings to environmental microbes in soil and sediment.

These findings reveal a previously unappreciated ecological function of bacterial chemotaxis. Chemotaxis is traditionally understood as a foraging strategy for navigating toward nutrients and away from toxins or for promoting range expansion of a bacterial population (*12*–*17*). Our work identifies an additional ecological function: predator evasion through collective spatial dynamics, enabling bacterial populations to persist under intense predation pressure without the fitness costs of evolving genetic resistance. As quantified by the escape parameter $\tilde{\chi }$, bacterial strains with higher chemotactic efficiency or lower phage susceptibility are more likely to successfully colonize distant locations, helping to inform applications in bioremediation and industrial fermentation. Conversely, in the context of phage therapy, pathogenic bacteria could escape phage via collective migration through tissues, suggesting treatment strategies that account for spatial dynamics (e.g., combining phage with motility inhibitors or engineering phage with enhanced diffusivity through capsid modifications). More broadly, we conjecture that the interplay among distinct selective pressures for nutrient acquisition, territorial expansion, and predator evasion jointly shape the evolution of motility and chemotactic sensitivity across diverse bacterial species and environmental settings.

## MATERIALS AND METHODS

### Materials and cell culture

We use a strain of *E. coli* MG1655 that constitutively expresses GFP. T4 phage (American Type Culture Collection) was propagated using standard protocols to create a 10^11^ PFU/ml stock solution in SM buffer. Reagents were from Sigma-Aldrich unless noted.

### Preparation of phage-loaded granular hydrogels

We prepare granular hydrogel packings by mixing dry granules of crosslinked acrylic acid–based copolymers (Carbomer 980, Lubrizol, Piscataway, NJ) in liquid EZ Rich media (Teknova, Hollister, CA), as described previously (*27*, *29*, *31*, *36*). The defined medium contains L-serine, the primary chemoattractant, at a concentration of 10 mM. In addition, we supplement the medium with 10 mM CaCl_2_ and 10 mM MgCl_2_ following conventional protocol for enhanced phage infection efficiency. We use 10 M NaOH to adjust the final pH to 7.4 for optimal cell viability.

Using a stock solution of T4 phage in SM buffer (50 mM tris-HCl, 8 mM MgSO_4_, 100 mM NaCl, and 0.01% gelatin) at a concentration of 1 × 10^11^ PFU/ml, we prepare phage-loaded granular hydrogel packings by reducing the stock concentration 10-, 100-, or 1000-fold by gentle mixing in granular hydrogel media, resulting in final phage concentrations of 10^8^, 10^9^, and 10^10^ PFU/ml, respectively. Phage-loaded granular hydrogel packings are then mixed with a pipette tip or inoculum wand for 1 to 2 min to ensure uniform distribution. We prepare tightly packed granular hydrogel packings at a final polymer concentration of 0.6 wt % and loosely packed granular hydrogel packings at a final polymer concentration of 0.55 wt %.

### Rheological characterization of granular hydrogel packings

To characterize rheological properties, we load 2 ml of granular hydrogel between roughened parallel plates (50 mm in diameter) with a gap of 1 mm on an oscillatory shear rheometer (Anton Paar MCR301). We determine the storage (*G*′) and loss (*G*″) moduli under low strain (1%) at a frequency sweep of 10^−3^ to 100 Hz, and we perform a unidirectional shear rate ramp from 10^−2^ to 10^3^ s^−1^ to characterize yield stress behavior.

### Determining phage stability in granular hydrogels

To demonstrate the stability of T4 phage within granular hydrogels at 30°C, we prepare a phage-free and a phage-loaded granular hydrogel (10^9^ PFU/ml) and incubate it at 30°C for 24 hours. The next day, we dilute a stationary phase culture of *E. coli* [optical density (OD) ∼1.2] 100-fold into the preincubated phage-free and phage-loaded granular hydrogels. We also prepare fresh phage-free and phage-loaded granular hydrogels and dilute a stationary phase culture of *E. coli* 100-fold into freshly prepared granular hydrogels as well. Using a pipette tip, we gently mix *E. coli* into granular hydrogels to ensure uniform distribution. Using a plate reader (Agilent BioTek), we measure the OD at 600 nm in a 96-well plate every 10 min for 24 hours while incubating at 30°C. We compare bacterial growth in freshly prepared and preincubated phage-free granular hydrogels, as well as killing dynamics in freshly prepared and preincubated phage-loaded granular hydrogels. Preincubation at 30°C does not affect *E. coli* growth in phage-free granular hydrogels, nor does it affect phage-killing dynamics in phage-loaded granular hydrogels, demonstrating the stability of T4 phage in our granular hydrogel system at 30°C for at least 48 hours.

### Setting up chemotaxis assays in glass capillaries

To set up chemotaxis assays, we use square borosilicate glass capillaries (VitroCom) with an inner diameter of 3 mm cut to a length of 3 cm. We transfer ∼170 μl of phage-loaded granular hydrogel into the center of the capillary using a pipette. We prepare a dense suspension of *E. coli* by centrifuging 2 ml of an exponential phase culture (OD ∼ 0.6) made in 2 wt % LB media at 3000 rpm for 8 min, then subsequently removing the supernatant, and resuspending the bacterial pellet in 100 μl of EZ Rich media, resulting in a dense suspension with a concentration of ∼1 × 10^10^ CFU/ml. We transfer 10 μl of the dense cell suspension to the left-hand side of the glass capillary, creating a defined bacteria-granular hydrogel interface. We seal both sides of the glass capillary with ∼30 μl of oxygen-permeable paraffin oil to prevent evaporation. Filled glass capillaries are secured onto long microscope cover glass (74 mm by 32 mm) with parafilm and transferred to the confocal microscope for imaging.

### Confocal microscopy chemotaxis assays

To image bacterial migration in phage-loaded granular hydrogels, we use a Nikon A1R inverted laser-scanning confocal microscope with a stage-top warming plate set to 30°C. Over the length of the capillary, we take three vertical planar images with a step size of 100 μm. We take images every 20 min for up to 30 hours. For postprocessing, we take maximum intensity *z* projections and tile images across the length of the capillary for use in subsequent analysis.

### Analyzing chemotaxis

We use custom MATLAB scripts to track the position, width, and intensity of the chemotactic fronts over time. We do this by averaging GFP signal intensity in the direction perpendicular to chemotactic front migration across the length of the capillary, where the chemotactic front appears as a local peak in signal. We track peak location to map front position, peak width at half max to track front width, and normalized maximum peak signal to track front intensity over time.

### Bacterial growth and phage predation in well-mixed granular hydrogels

To assess *E. coli* growth in well-mixed environments, we dilute a stationary phase culture of *E. coli* (OD, ∼1.2) prepared in 2 wt % LB by 100-fold into phage-free and phage-loaded granular hydrogels (10^8^ to 10^10^ PFU/ml). We gently mix with a pipette to ensure uniform distribution. Using a plate reader (Agilent BioTek), we measure the OD at 600 nm in a 96-well plate every 10 min for 24 hours while incubating at 30°C.

### Assaying for phage resistance in chemotactic fronts

To assess the possibility of phage resistance in chemotactic fronts, we prepare chemotactic front assays in glass capillaries and incubate at 30°C until chemotactic fronts have moved a distance of ∼1 cm. This occurs after ∼7 hours for phage-free and 10^8^ PFU/ml T4 concentrations, ∼11 hours for 10^9^ PFU/ml T4 concentrations, and ∼19 hours for 10^10^ PFU/ml T4 phage concentrations. Chemotactic waves are visible in the capillaries by eye. To prepare cell stocks of bacteria isolated from chemotactic fronts, we use a pipette to remove 5 μl of granular hydrogel at the location of the chemotactic front, dilute into 16% glycerol stocks, and freeze at −80°C for future use.

### Determining phage diffusion coefficients in granular hydrogels

To determine the diffusion coefficient of T4 phage in our granular hydrogels, we fluorescently label T4 phage with SYBR Gold stain (Thermo Fisher Scientific) by mixing 1 ml of phage stock solution with 1 μl of SYBR Gold. We incubate at 4°C for 30 min in a dark environment, then subsequently add 2 ml of purification buffer (20 wt % PEG-8000, 2 M NaCl), and incubate at 4°C overnight to crash phage out of solution.

### Statistical analysis

We report all data as mean ± SD, unless otherwise indicated. We conduct statistical analysis in GraphPad Prism 9 using one-way analysis of variance (ANOVA) and a Tukey’s post hoc comparison, unless otherwise indicated. For all samples, *n* ≥ 3, *P < 0.05, **P < 0.01, ***P < 0.001, and ****P < 0.0001; ns indicates not significant.

### Nondimensionalization of the model

To reduce the number of parameters of our model, we scale all variables using the following scales for length, time, bacterial density, nutrient and chemoattractant concentration, and phage density, respectively

$$\begin{array}{c} \ell_{c}=\sqrt{\frac{{\mathrm{Da}}_{\text{init}}}{{\mathrm{kb}}_{s,\text{init}}}},\;t_{c}=\frac{\ell_{c}^{2}}{\chi }=\frac{{\mathrm{Da}}_{\text{init}}}{\chi {\mathrm{kb}}_{s,\text{init}}},\\ b_{c}=b_{s,\text{init}},\;c_{c}=c_{\text{init}},\;a_{c}=a_{\text{init}},\;p_{c}={\left(\gamma_{s}t_{c}\right)}^{-1} \end{array}$$ (8)

We use the initial bacterial concentration of susceptible bacteria, $b_{s,\text{init}}$ to scale all bacterial density fields. The emerging dimensionless parameters as well as the values of both model parameters and dimensionless parameters used in simulations are given in Table 1.
