# Supplementary material for: Death and chemotaxis: Bacterial chemotaxis enables collective escape from phage predation
Source: Sci Adv. 2026 Oct 16;12(42):eaeg2358. doi: 10.1126/sciadv.aeg2358 (PMC13674483; doi:10.1126/sciadv.aeg2358)
Supplement: Supplementary file 1 [file sciadv.aeg2358_sm.pdf]

Science Advances
AAAS

Supplementary Materials for

Death and chemotaxis: Bacterial chemotaxis enables collective escape from phage predation

Victoria G. Muir *et al.*

Corresponding author: Victoria G. Muir, vmuir@udel.edu; Alejandro Martínez-Calvo, amcalvo@princeton.edu; Ned S. Wingreen, wingreen@princeton.edu; Sujit S. Datta, ssdatta@caltech.edu

Sci. Adv. **12**, eaeg2358 (2026)
DOI: 10.1126/sciadv.aeg2358

The PDF file includes:

Legends for movies S1 to S5
Figs. S1 to S12

Other Supplementary Material for this manuscript includes the following:

Movies S1 to S5

SUPPLEMENTARY MATERIAL

Video S1. Representative confocal microscopy time lapse video of chemotactic front formation along the capillary for a control case (no phage, top) and increasingly high phage concentration (10^8 - 10^{10} pFU/mL). Scale bar = 1 mm.

Video S2. Normalized model number densities of bacterial and phage populations, and chemoattractant concentration as functions of position x over time for an initial phage concetration of 10^8 PFU/mL.

Video S3. Same as Video S2 but for initial phage concentration 10^9 PFU/mL.

Video S4. Same as Video S2 but for initial phage concentration 10^{10} PFU/mL.

Video S5. Representative confocal microscopy time lapse video of chemotactic front formation along the capillary for a control case (no phage, top) and intermediate phage concentration (10^9 PFU/mL) in loosely packed granular hydrogel. Scale bar = 1 mm.

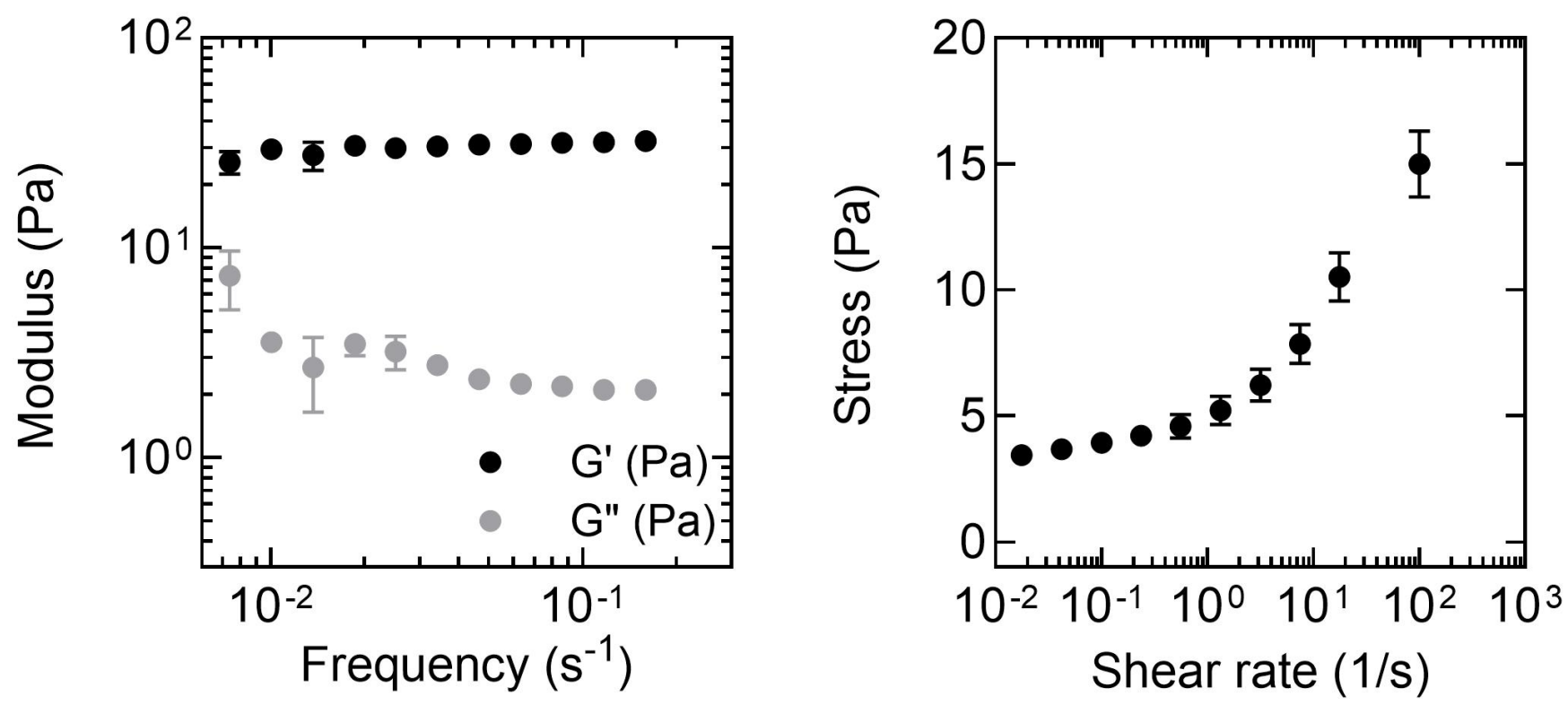

Fig. S1. **Rheological characterization of granular hydrogels (0.6 wt.% Carbopol).** Left: Storage (G') and loss (G") moduli as a function of frequency (10^{-3} - 1 Hz). Right: Stress as a function of shear rate (10^{-2} – 10^3 s^{-1}). Data represent mean $\pm$ standard deviation ($n \geq 3$).

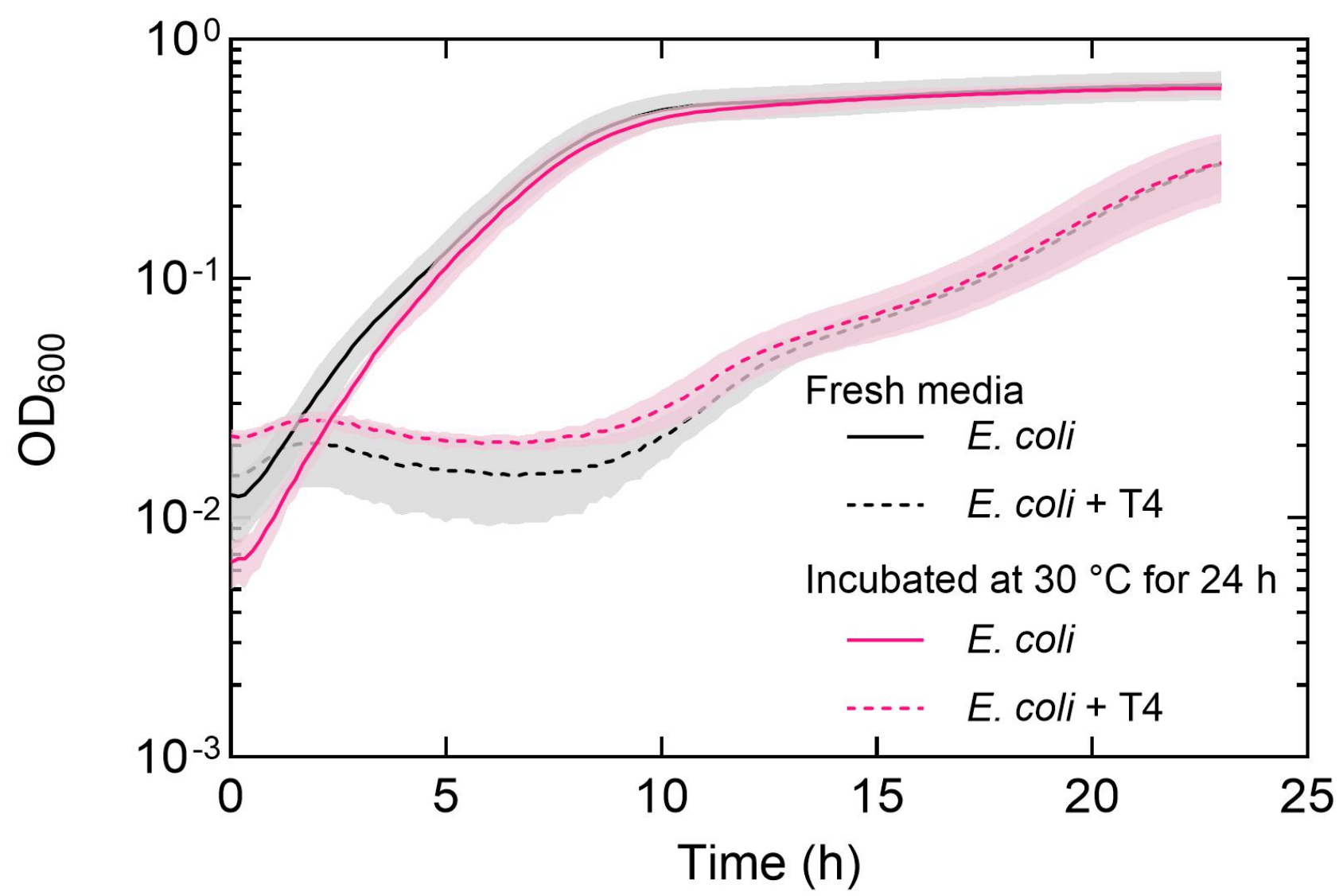

Fig. S2. **Phage stability in granular hydrogels.** Optical density at 600 nm (OD600) of *E. coli* growing in granular hydrogels (0.6 wt.% Carbopol) with (dotted) or without (solid) T4 phage. Black curves show OD600 over 24 hours after inoculating fresh granular hydrogels (+/- phage) with *E. coli*. Pink curves show OD600 over 24 hours after inoculating 24-hour-old hydrogels (+/- phage) incubated at 30°C. Growth (no phage) and killing dynamics (with phage) remain unchanged after hydrogels are incubated for 24 hours. We conclude that phage remain stable in granular hydrogels for at least 24–48 hours, covering the duration of experiments in this study. Data represent mean $\pm$ standard deviation ($n \geq 3$).

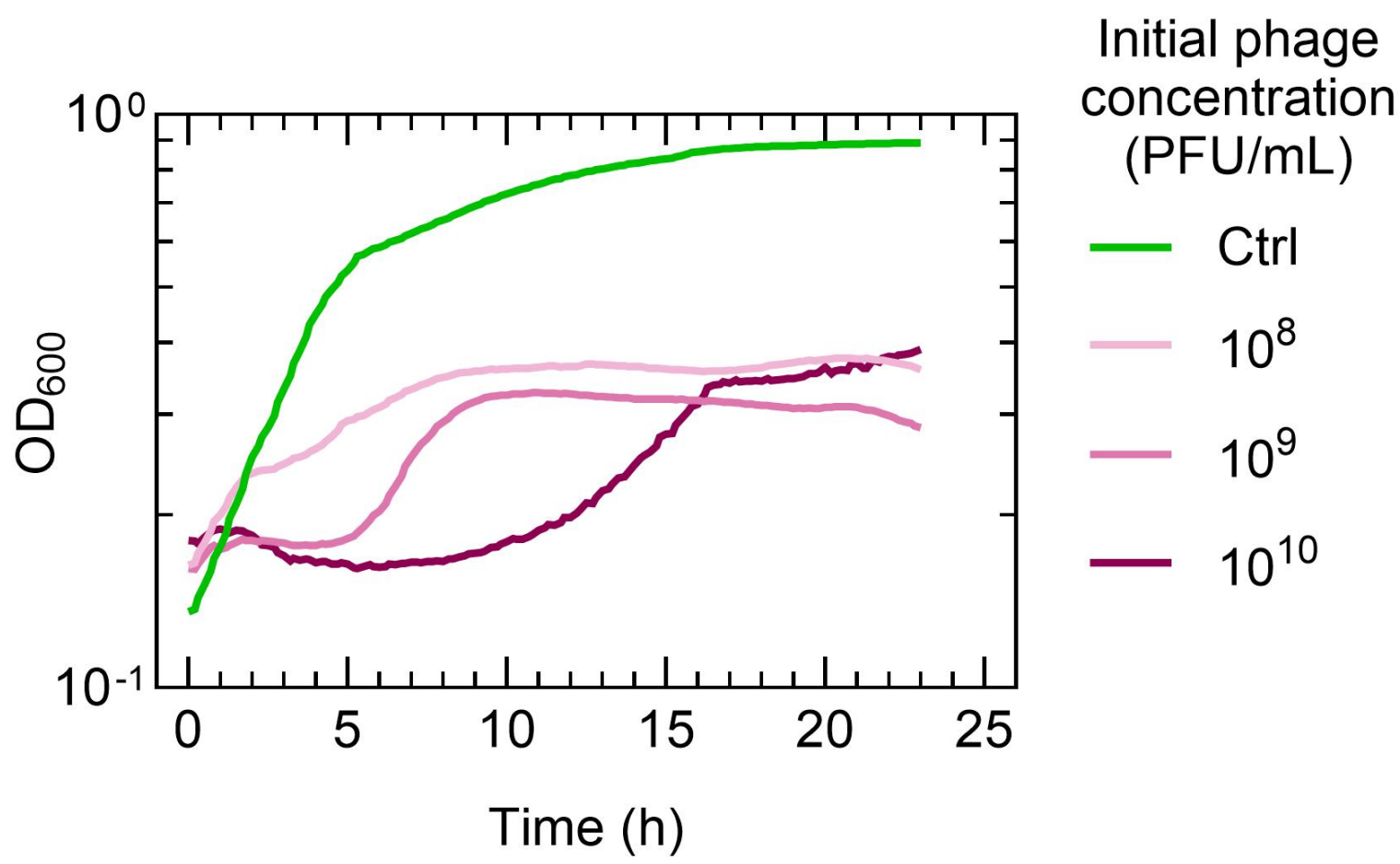

Fig. S3. **Optical density at 600 nm (OD600) of *E. coli* growing in hydrogels (0.6 wt.% Carbopol) at various phage concentrations in a well-mixed environment.** $n = 1$.

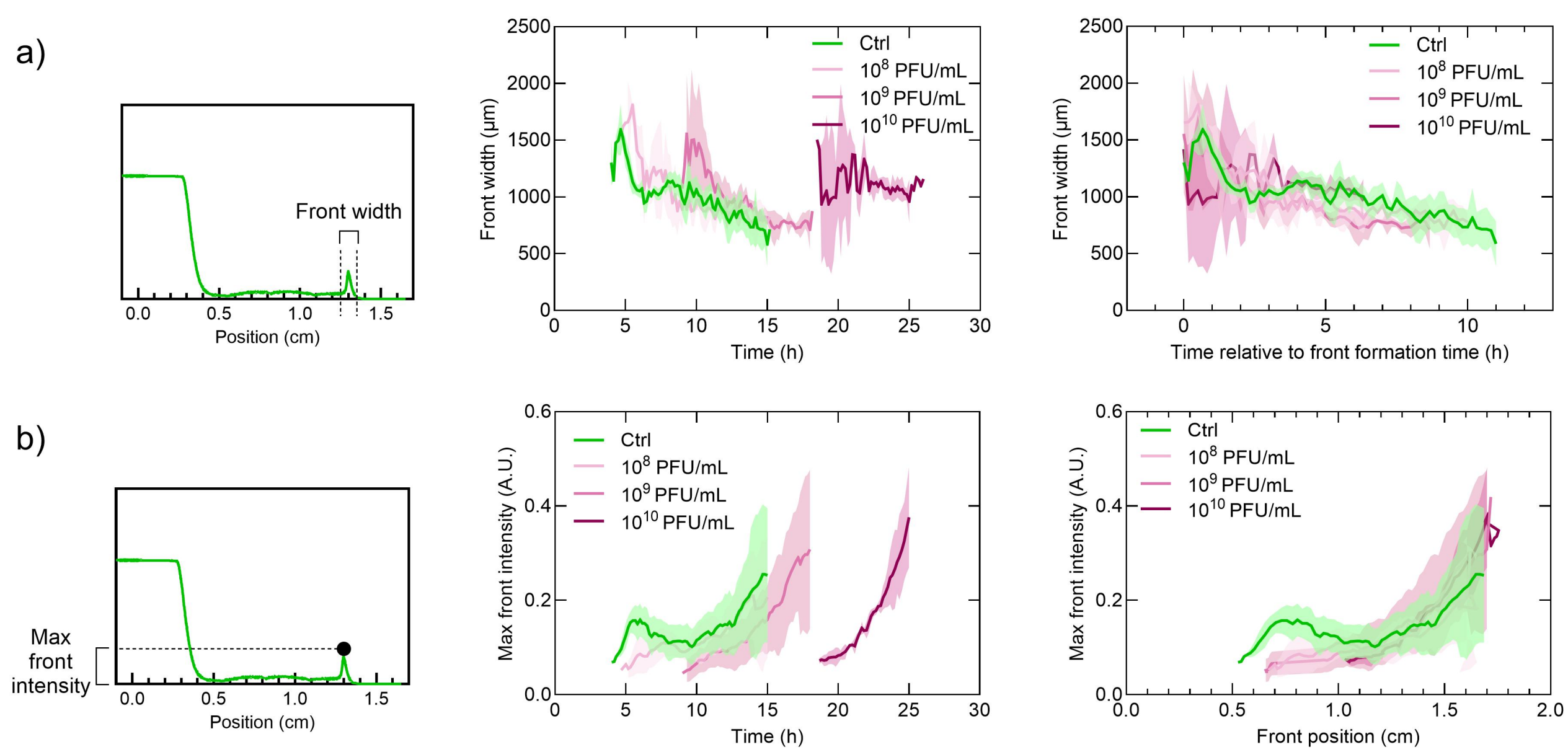

Fig. S4. **Characterizing front width and front intensity over time.** (**a**) Width of chemotactic fronts over time at various phage concentrations. We plot the width as a function of time and relative to the initial front formation time. (**b**) Maximum fluorescence intensity of chemotactic fronts over time, plotted as a function of time and as a function of front position. Data represent mean ± standard deviation ($n \geq 3$).

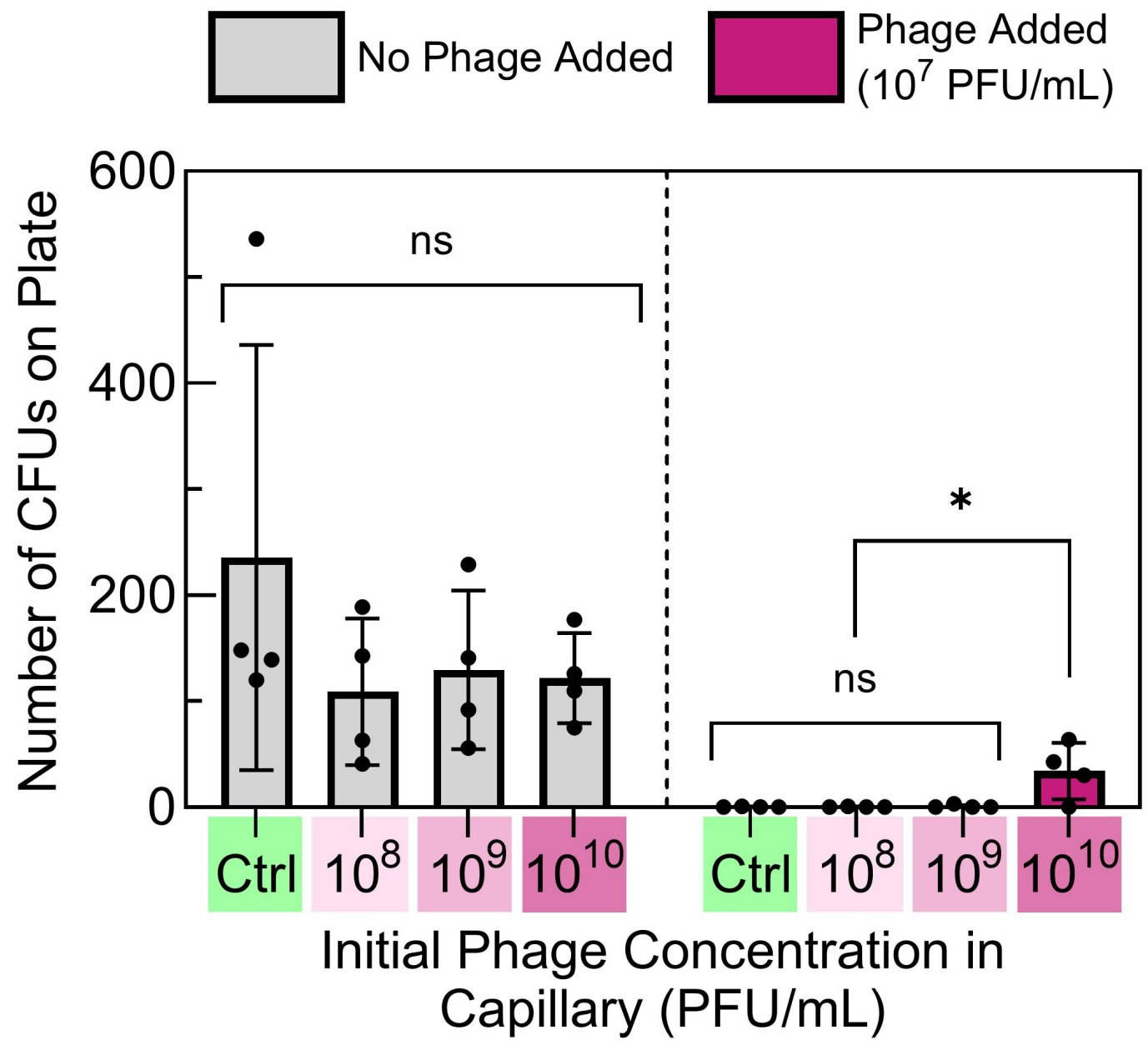

Fig. S5. **Total colony-forming units (CFUs) isolated from chemotactic fronts formed in the presence of phage.** Total CFUs from chemotactic fronts plated on soft agar without phage (light gray) and with phage (dark pink, 10^7 PFU/mL). Data represent mean ± standard deviation ($n \geq 4$). We use one-way ANOVA to determine statistical significance: ns = not significant, $*p < 0.05$.

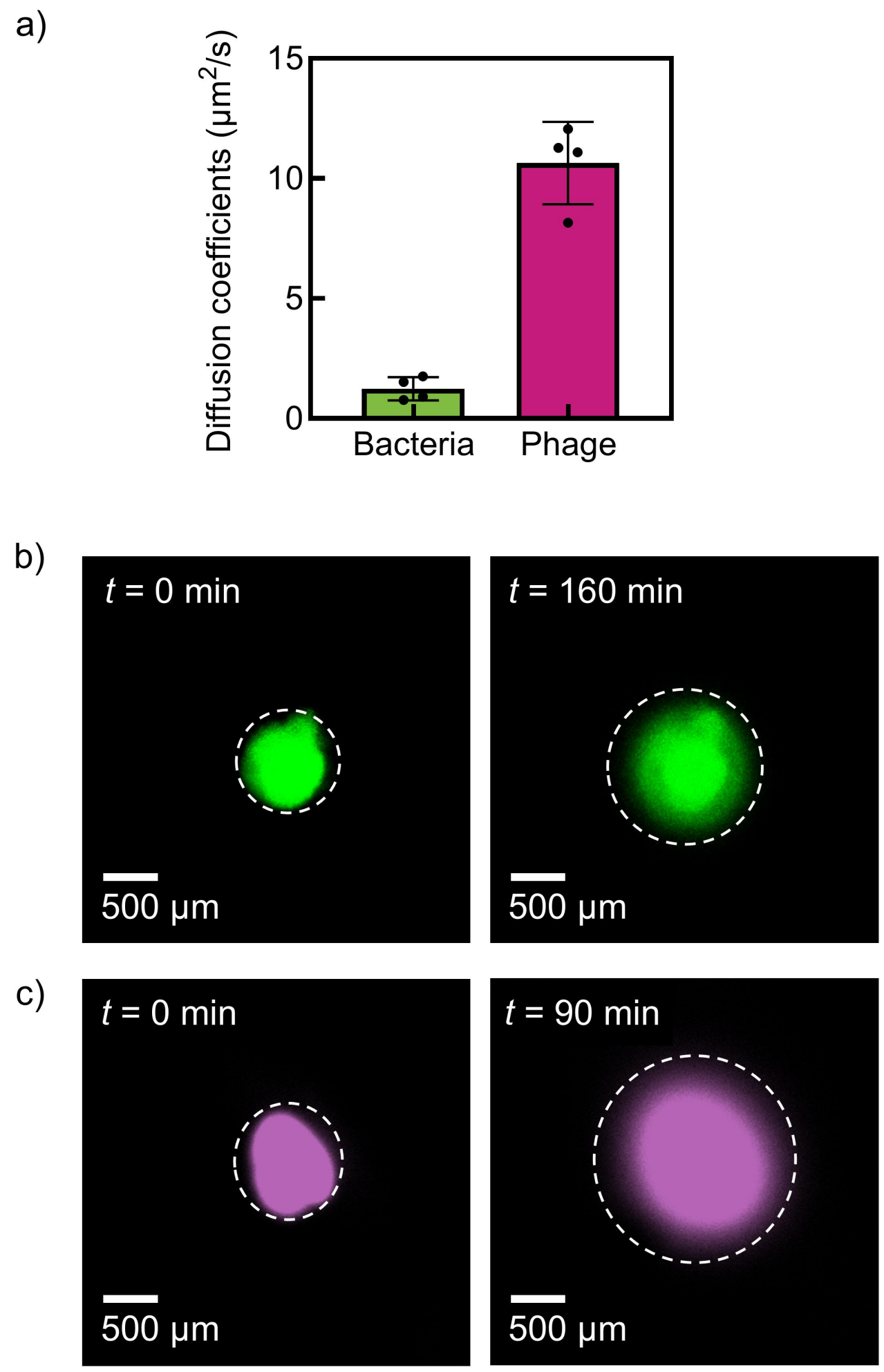

Fig. S6. **Diffusion coefficients of motile bacteria and phage in granular hydrogels (0.6 wt.% Carbopol).** (**a**) Diffusion coefficients of bacteria and phage measured in granular hydrogels experimentally. (**b**) Representative maximum projection confocal z-stack images of bacteria diffusing in granular hydrogels at $t = 0$ min and $t = 160$ min (prior to chemotactic front formation). Scale bar = 500 μm. (**c**) Representative maximum projection confocal z-stack images of fluorescently labeled phage diffusing in granular hydrogels at $t = 0$ min and $t = 90$ min. Scale bar = 500 μm. Data represent mean $\pm$ standard deviation ($n \geq 4$).

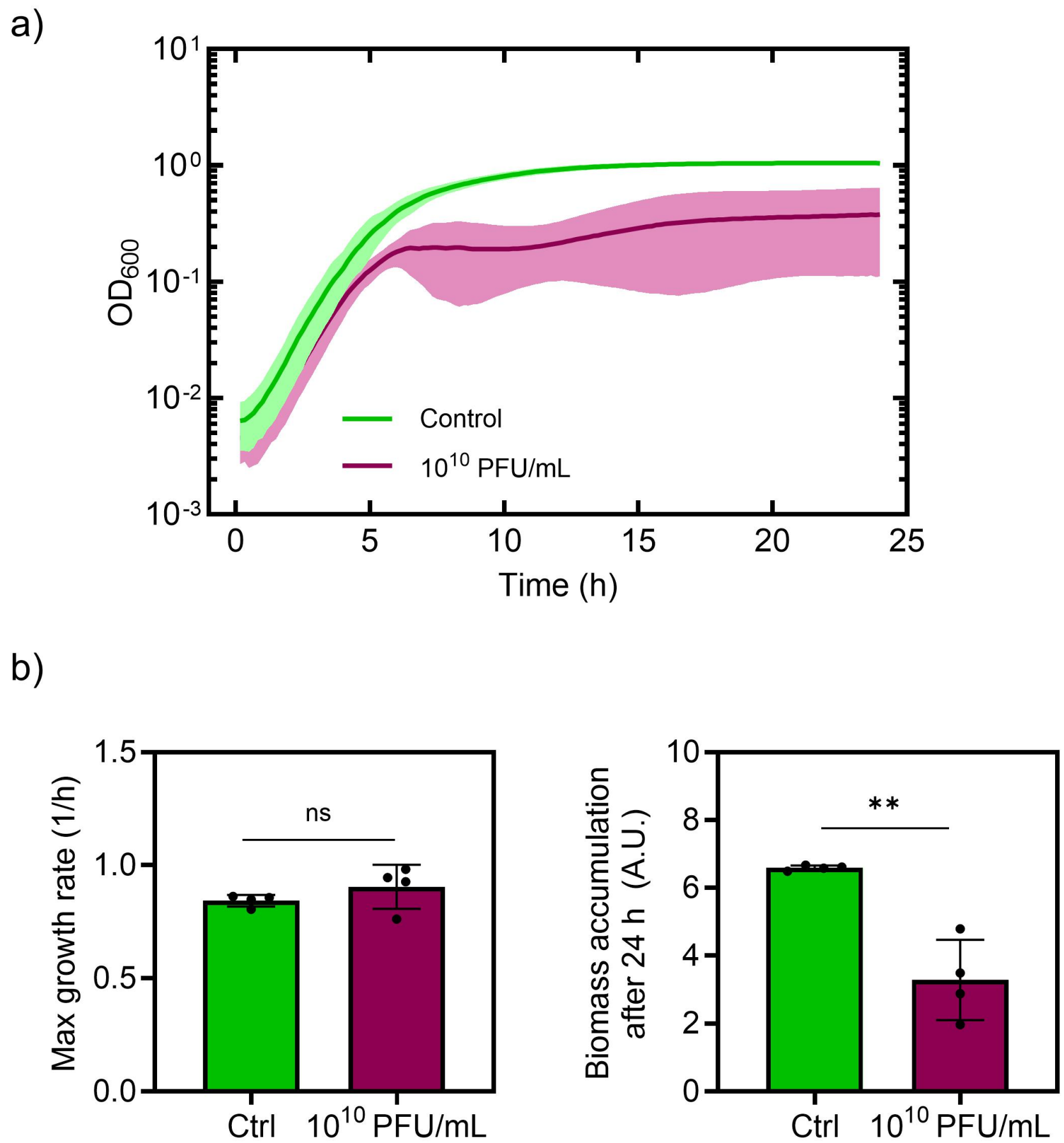

Fig. S7. **Growth reduction of *E. coli* less susceptible to phage infection.** (**a**) Optical density at 600 nm (OD600) of *E. coli* isolated from chemotactic fronts formed without phage (green) and *E. coli* isolated from chemotactic fronts formed in the presence of 10^{10} PFU/mL phage (pink). (**b**) Quantification of maximum growth rate and biomass accumulation (area under the curve) after 24 h for *E. coli* isolated from chemotactic fronts. Data represent mean ± standard deviation ($n \geq 3$). We use one-way ANOVA to determine statistical significance: ns = not significant, $^{**}p < 0.01$.

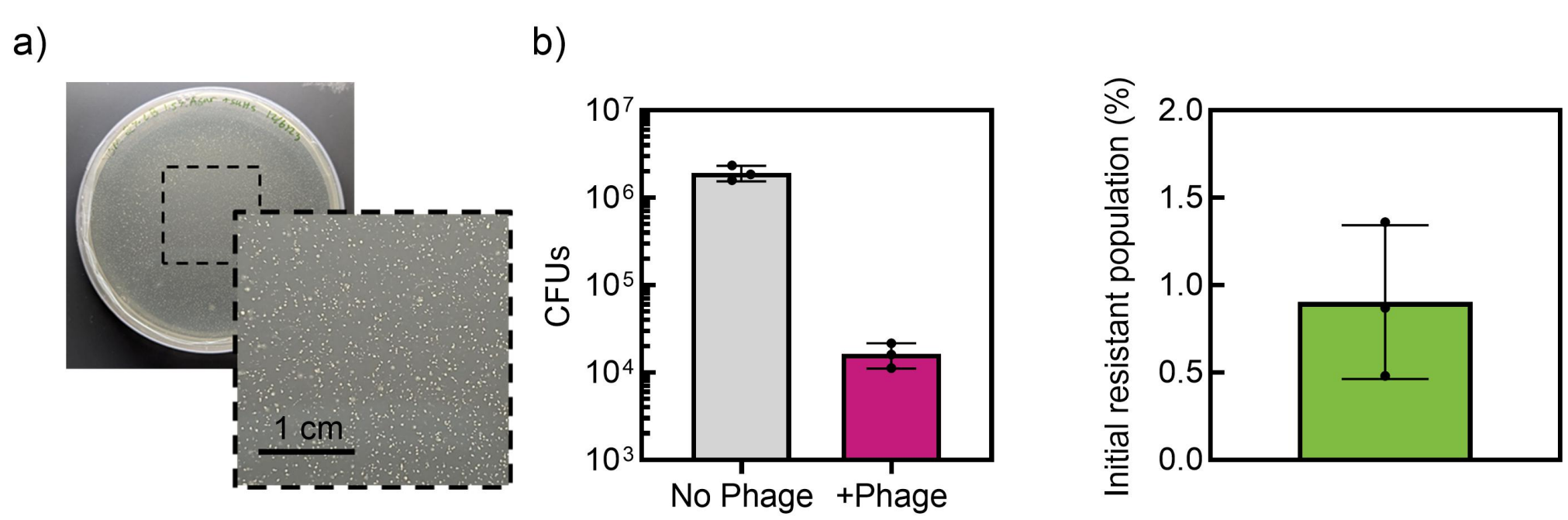

Fig. S8. **Initial fraction of phage-resistant *E. coli* in stock solutions.** (**a**) Representative image of CFUs from stock solution growing on agar plates with T4 phage. (**b**) Quantification of total CFUs from stock solutions plated on phage-free (grey) and phage-rich plates (pink), and the percentage of phage-resistant bacteria in the stock population. Data represent mean ± standard deviation ($n \geq 3$).

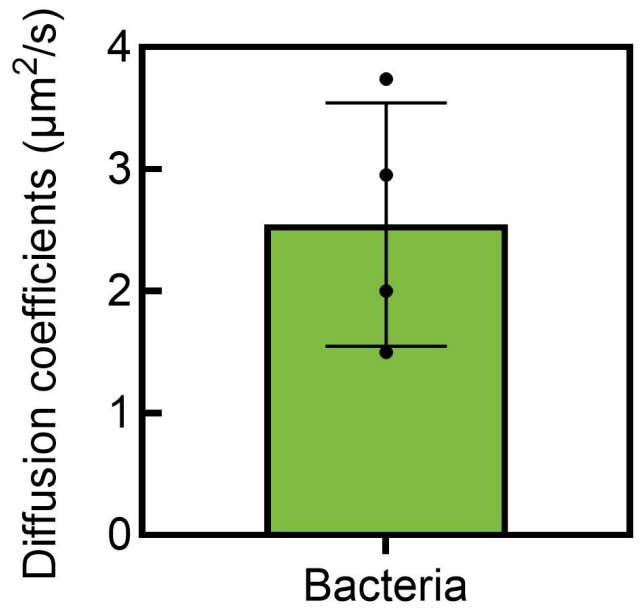

Fig. S9. **Diffusion coefficients of bacteria in measured experimentally in granular hydrogels (0.55 wt.% Carbopol).** ($n \geq 4$).

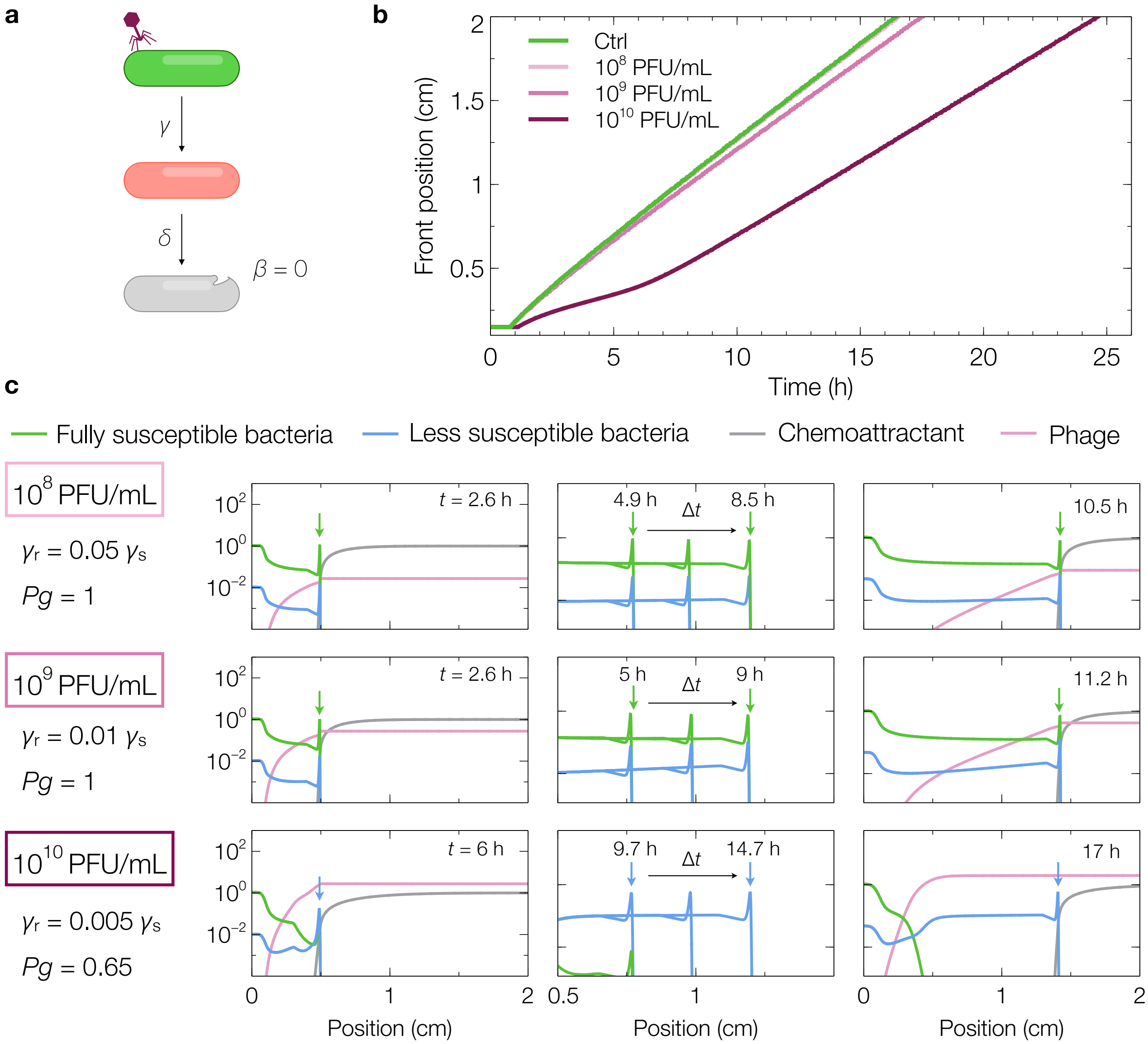

Fig. S10. **Phage burst size β influences bacterial chemotactic front formation dynamics.** **(a)** Schematic of a modified model (relative to the one presented in the main text and Fig. 3a) in which phages can infect and kill bacteria but do not proliferate via bursting, implying $\beta = 0$. **(b)** Bacterial chemotactic front position as a function of time in a phage-free environment and for $p_{\text{init}} = 10^8$, 10^9, and 10^{10} PFU/mL, from the model with $\beta = 0$, i.e., non-bursting phages. In this modified model, the chemotactic front is appreciably delayed only for $p_{\text{init}} = 10^{10}$, implying that the phage burst size β alters the phase boundaries shown in Fig. 4. **(c)** Normalized concentration profiles of bacteria, chemoattractant, and phage at different times for various phage concentrations. Bacteria and chemoattractant profiles are normalized by their initial concentrations at $t = 0$, and the phage profile is normalized by the characteristic concentration $\chi/(\gamma_s \ell_c^2)$, where ℓ_c is the chemoattractant penetration scale (see Table 1 and Methods). Initially, nutrient and chemoattractant are uniform across the domain with concentrations c_{init} and a_{init}, respectively. Bacteria are uniformly distributed in a phage-free region ($0 < x < x_{\text{init}}$) adjacent to uniform phage suspension ($x_{\text{init}} < x < L$), forming a slab-shaped colony facing the phage-rich environment as in the experiments. Here, x_{init} denotes the position of the bacteria–phage contact interface at $t = 0$, and L is the domain length. We assume that phage are stable and long-lived over the experimental duration and do not burst ($\beta = 0$), in contrast to the results shown in Fig. 3. Arrows indicate chemotactic front position.

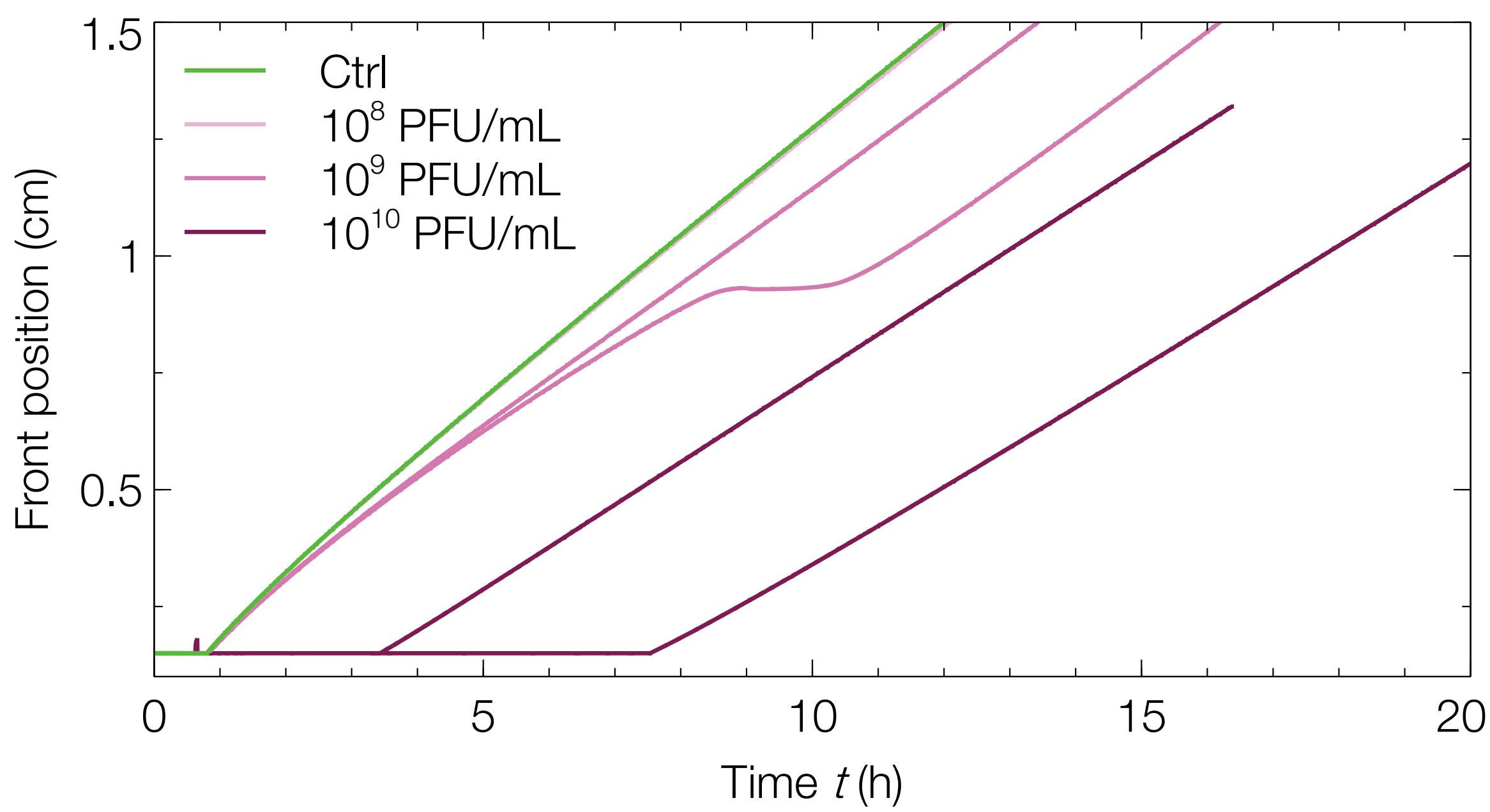

Fig. S11. **The ratio of susceptible to less susceptible cells affects the time required for a chemotactic front to form, but not its steady-state speed.** Bacterial chemotactic front position as a function of time for a phage-free control and for $p_{\text{init}} = 10^8$, 10^9, and 10^{10} PFU/mL, obtained from the model for an initial fraction of 1% less susceptible cells (solid curves), corresponding to the results shown in the main text, and 5% less susceptible cells (dashed curves).

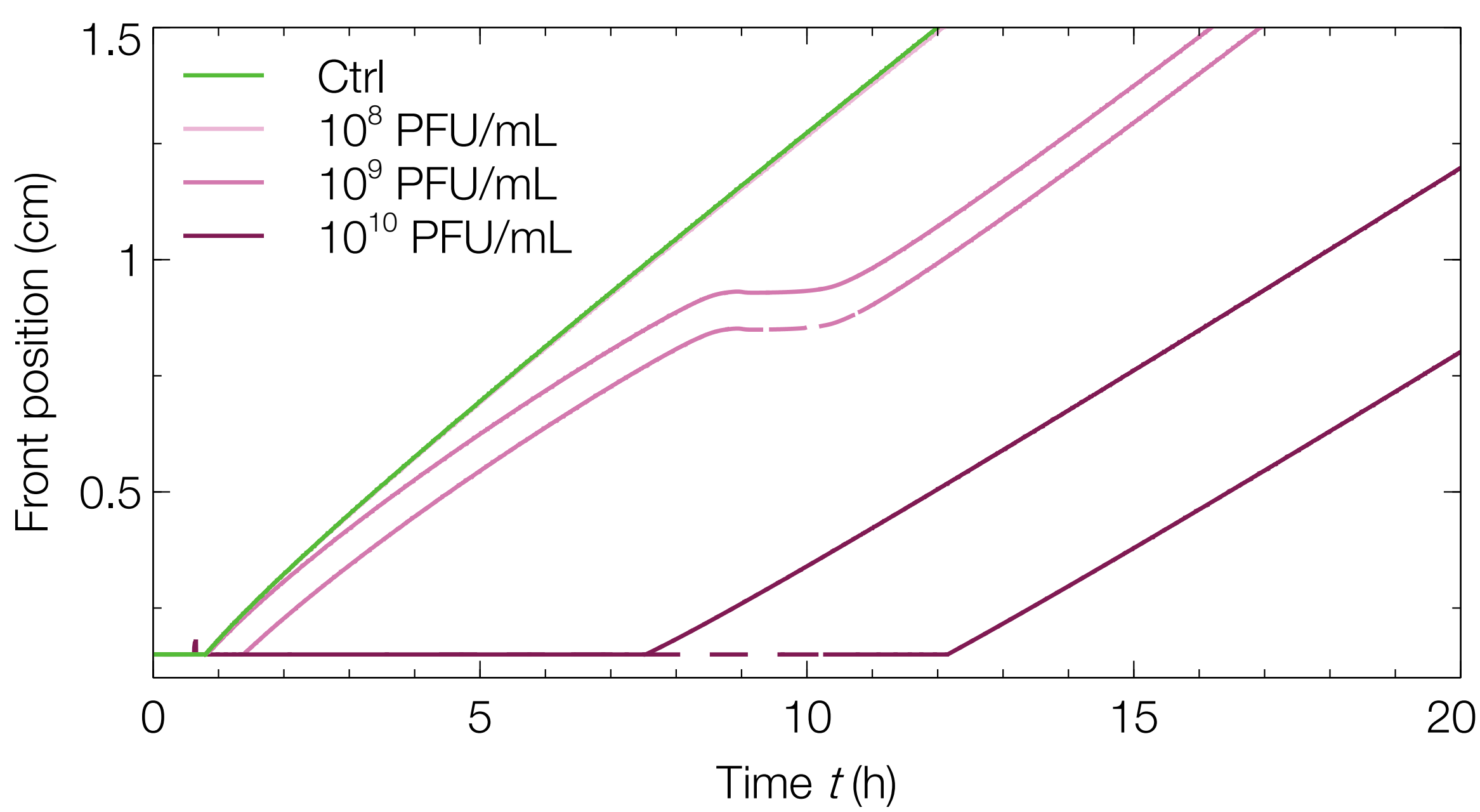

Fig. S12. **The size of the bacterial inoculum affects the time required for a chemotactic front to form, but not its steady-state speed.** Bacterial chemotactic front position as a function of time for a phage-free control and for $p_{\text{init}} = 10^8$, 10^9, and 10^{10} PFU/mL, obtained from the model for an initial size of $x_{\text{init}} = 1$ mm, corresponding to the results shown in the main text, and a smaller size $x_{\text{init}} = 0.25$ mm (dashed curves).
